# Haplotype-based association mapping of genomic regions associated with *Zymoseptoria tritici* resistance using 217 diverse wheat genotypes

**DOI:** 10.1186/s12870-024-05400-1

**Published:** 2024-07-18

**Authors:** Magdalena Radecka-Janusik, Urszula Piechota, Dominika Piaskowska, Piotr Słowacki, Sławomir Bartosiak, Paweł Czembor

**Affiliations:** Plant Breeding and Acclimatization Institute – National Research Institute, Radzików, Błonie, 05-870 Poland

**Keywords:** Genome-wide association study, Haploblocks, Septoria tritici blotch, Resistance, Wheat

## Abstract

**Background:**

Septoria tritici blotch (STB) is considered to be one of the most destructive foliar wheat diseases and is caused by *Zymoseptoria tritici*. The yield losses are severe and in Northwestern Europe can reach up to 50%. The efficacy of fungicides is diminishing due to changes in the genetic structure of the pathogen. Therefore, resistance breeding is the most effective strategy of disease management. Recently, genome-wide association studies (GWAS) have become more popular due to their robustness in dissecting complex traits, including STB resistance in wheat. This was made possible by the use of large mapping populations and new sequencing technologies. High-resolution mapping benefits from historical recombination and greater allele numbers in GWAS.

**Results:**

In our study, 217 wheat genotypes of diverse origin were phenotyped against five *Z. tritici* isolates (IPO323, IPO88004, IPO92004, IPO86036 and St1-03) and genotyped on the DArTseq platform. In polytunnel tests two disease parameters were evaluated: the percentage of leaf area covered by necrotic lesions (NEC) and the percentage of leaf area covered by lesions bearing pycnidia (PYC). The disease escape parameters heading date (Hd) and plant height (Ht) were also measured. Pearson’s correlation showed a positive effect between disease parameters, providing additional information. The Structure analysis indicated four subpopulations which included from 28 (subpopulation 2) to 79 genotypes (subpopulation 3). All of the subpopulations showed a relatively high degree of admixture, which ranged from 60% of genotypes with less than 80% of proportions of the genome attributed to assigned subpopulation for group 2 to 85% for group 4. Haplotype-based GWAS analysis allowed us to identify 27 haploblocks (HBs) significantly associated with analysed traits with a *p*-value above the genome-wide significance threshold (5%, which was –log10(*p*) > 3.64) and spread across the wheat genome. The explained phenotypic variation of identified significant HBs ranged from 0.2% to 21.5%. The results of the analysis showed that four haplotypes (HTs) associated with disease parameters cause a reduction in the level of leaf coverage by necrosis and pycnidia, namely: Chr3A_HB98_HT2, Chr5B_HB47_HT1, Chr7B_HB36_HT1 and Chr5D_HB10_HT3.

**Conclusions:**

GWAS analysis enabled us to identify four significant chromosomal regions associated with a reduction in STB disease parameters. The list of valuable HBs and wheat varieties possessing them provides promising material for further molecular analysis of resistance loci and development of breeding programmes.

**Supplementary Information:**

The online version contains supplementary material available at 10.1186/s12870-024-05400-1.

## Background

Wheat (*Triticum aestivum* L.) is considered to be one of the most important staple crops. According to data published by the Food and Agriculture Organization of the United Nations [1], in the 2014–2020 period wheat products provided approximately 38% of the daily calorie intake among Europeans and 24% in the world. In 2021, wheat was the most widely produced cereal in Europe (256 million tonnes (mt) annually) and second most overall (749 mt) [2]. Nevertheless, to meet the food demand of a rising world population, it is necessary to achieve a 25%–70% production increase by 2050 above the 2014 production baseline [3]. In general, future production growth will come from yield growth, rather than increasing the intensity and area of harvest [3].

One strategy to increase production is minimizing yield losses due to diseases. Septoria tritici blotch (STB) is one of the most serious threats to European wheat. It is a foliar disease caused by *Zymoseptoria tritici* (Desm.) (teleomorph *Mycosphaerella graminicola*, syn. *Septoria tritici*). The fungus spreads either by wind or by rain splash [4] and therefore its symptoms are the most severe in temperate climates with high humidity and frequent rainfall [5]. According to McDonald and Linde [6] *Z. tritici* is designated as a high-risk pathogen due to its high adaptation potential and large effective population size. *Z. tritici* is ranked seventh in the list of the top ten fungal plant pathogens according to their economic and scientific impact [7]. Dean and co-workers [7] noted huge genetic variation of *Z. tritici* isolates observed within a single field as well as fast pathogen evolution under selection pressure. Recently, Savary et al. [8] reported that in Northwestern Europe it causes up to 5.5% yield loss, despite fungicide usage. However, STB epidemics can be far more devastating if the crop is not properly managed or due to unfavourable weather conditions (wind and high humidity, especially in May and June). STB may cause up to 50% yield loss, especially if the top leaves become infected, as their photosynthesis contributes greatly to grain filling [9, 10]. Furthermore, Z*. tritici* populations have been reported to be able to develop resistance to diverse classes of fungicides [11–15]. This can happen even within a single season [16]; therefore effective fungicide protection options become more limited [17]. This suggests that disease management should rely on host resistance with reduced use of fungicides considering negative environmental effects.

Resistance to STB in wheat is complex and can be governed by major genes offering a quasi-qualitative response, as well as by quantitative trait loci (QTL) that typically encompass several genes with minor to moderate phenotypic effects. Despite STB being one of the most destructive wheat diseases, only 23 major resistance genes have been identified, predominantly in bread wheat [18–20]. Conversely, over 160 QTL conferring STB resistance have been identified. Quantitative resistance is perceived as polygenic and generally exerts a smaller effect than R genes, making it more durable due to reduced selection pressure on fungal populations [18, 21–23]. Under both controlled and filed conditions, STB resistance appears quantitative, largely additive, and exhibits varied heritability [18]. This suggests that disease management should not rely solely on the use of fungicides. Instead, we should explore other alternatives, including genetic resistance.

All 23 identified major STB resistance genes as well as most QTL were characterized and mapped with linkage mapping in bi-parental populations [18, 19]. Such an approach is highly time-consuming. The evaluation of progeny needs to be preceded by crosses and derivation of mapping generations or creation of doubled haploids [24]. Another approach, genome-wide association studies (GWAS) on a diverse genotype panel, avoids the crossing process and mapping progeny propagation. It benefits from historical recombination and greater allele numbers in GWAS, enhancing association analysis through LD decay [25]. GWAS has been employed to identify significant loci involved in STB resistance response [25–28]. Nevertheless there are some limitations of association studies based on a single SNP (single nucleotide polymorphism) array. SNPs provide only bi-allelic markers, and significantly associated SNPs may not represent the true causative locus [29]. Haploblock calling based on linkage disequilibrium followed by GWAS is one approach for overcoming the limitations of SNP analysis [30, 31]. Haplotypes within haploblocks consist of combinations of bi-allelic, co-inherited SNPs. Association analysis based on haploblocks takes into account the ancient recombination and increases the resolution of the region of interest [31].

Due to the observed rapid breakdown of existing resistance, it is important to support breeders with novel resistance genes effective against *Z. tritici* populations. It was reported that *Stb6* and *Stb16q*, which were once used as a source of resistance in European cultivars, have since lost their effectiveness in certain geographical regions [32]. A significant contribution to STB management can be provided by identification of resistance sources and resistance accumulation in breeding materials. Pyramiding quantitative resistance genes with additive effects is an approach to slow down the breakdown of resistance to pathogens, as it requires changes in multiple loci within the pathogen’s genome.

Different panels of wheat genotypes have already been investigated for resistance to STB by various teams of researchers using the GWAS method [33–36]. GWAS analysis based on haplotypes has been conducted in previous research [21, 37].

Our study is based on 217 diverse wheat genotypes phenotyped against five *Z. tritici* isolates and genotyped on the DArTseq platform. To obtain valuable information about significantly associated loci, we conducted haploblock calling followed by GWAS analysis. The objective of our study was to identify loci significantly associated with STB resistance in wheat.

## Results

### Phenotypic analysis

For each *Z. tritici* isolate, two data sets were produced: one for the NEC parameter, which was the percentage of leaf area covered by necrosis, and one for the PYC parameter – the area covered by pycnidia. Summarized information about all 20 resultant data sets is shown in Table 1 and detailed information – in Table S1. Observed disease parameters reveal a broad range of the coefficient of variation, from 32.47% to 115.38%.

**Table 1:** Statistical summary of phenotypic data produced for this study

No	Data set^a^	Population min. (%)	Population max. (%)	Population mean (%)	F value	CV^b^ (%)	Broad-sense heritability
1	IPO323_NEC	1.01	82.19	22.56	5.53	77.52	0.82
2	IPO323_PYC	0.00	68.52	10.30	3.21	112.39	0.69
3	IPO323_Ht	50.00	125.00	82.76	4.95	11.81	0.80
4	IPO323_Hd	146.00	159.00	153.33	6.16	1.30	0.84
5	IPO88004_NEC	0.79	98.97	29.46	4.38	69.46	0.77
6	IPO88004_PYC	0.02	88.29	17.09	4.07	98.37	0.75
7	IPO88004_Ht	45.00	105.00	65.97	1.14	13.72	0.86
8	IPO88004_Hd	156.00	177.00	172.03	22.71	1.93	0.96
9	IPO92006_NEC	6.14	99.77	57.55	3.91	35.09	0.74
10	IPO92006_PYC	0.22	92.65	34.16	3.11	62.44	0.68
11	IPO92006_Ht	20.00	100.00	66.90	2.17	13.24	0.54
12	IPO92006_Hd	149.00	166.00	158.85	6.33	2.04	0.84
13	IPO86036_NEC	5.82	99.52	53.22	1.95	43.25	0.49
14	IPO86036_PYC	0.00	53.20	8.17	2.82	115.38	0.65
15	IPO86036_Ht	50.00	120.00	82.08	-^c^	12.60	-
16	IPO86036_Hd	141.00	155.00	147.66	13.92	1.42	0.93
17	St1-03_NEC	18.33	99.82	57.56	1.43	32.47	0.30
18	St1-03_PYC	0.00	47.90	6.64	2.24	114.25	0.55
19	St1-03_Ht	50.00	120.00	83.80	-	12.14	-
20	St1-03_Hd	139.00	156.00	147.78	10.59	1.65	0.91

^a^Data set name consists of the following designations: isolate name, the percentage of leaf area covered by necrotic lesions (NEC) or the percentage of leaf area covered by lesions bearing pycnidia (PYC), plant height (Ht) or heading date (Hd); all values are significant at *p* < 0.01

^b^coefficient of variation

^c^data not available

**Fig. 1:**
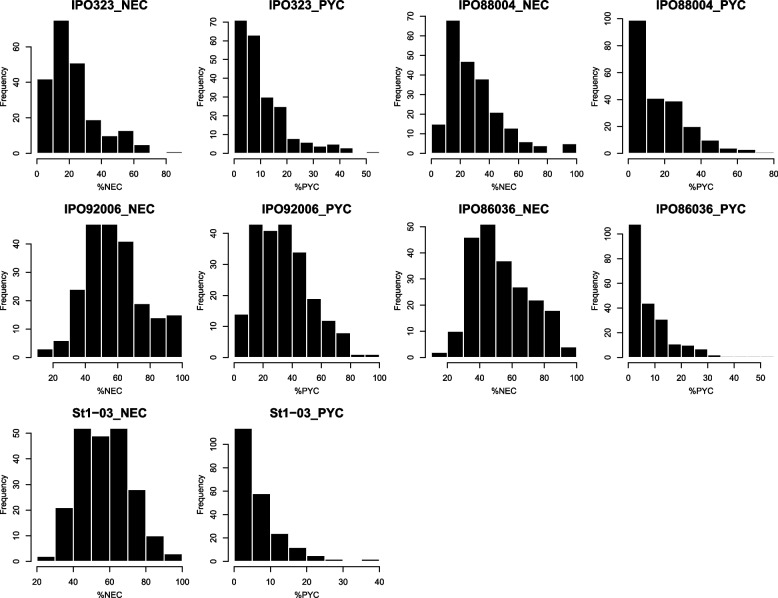
Frequency distribution of disease parameters in the 217 wheat genotypes set tested against to five *Z. tritici* isolates in the adult growth phase; NEC – percentage of leaf covered by necrosis; PYC – percentage of leaf covered by pycnidia

The wheat set demonstrated continuous distribution of disease parameter scores, but not every parameter showed normal distribution (Fig. 1). Broad-sense heritability for disease parameters was in the range 0.30–0.82 (Table 1); it proved that the variation observed is indeed due to genetic factors, and therefore the obtained phenotypic data are suitable for GWAS or QTL analysis. For disease escape traits, heading date and plant height, the value of *h*^*2*^ ranged from 0.54 to 0.96 (Table 1). Additionally, to estimate the relationship between necrosis and pycnidia coverage, Pearson’s correlation coefficient was calculated. It was significant, positive, and rather strong, ranging from 0.35 to 0.81 (Table 2). For plant height and heading date Pearson’s correlation was rather weak and mostly not significant (Table 2).

**Table 2:** Pearson’s correlation coefficient values between percentage of leaf area covered by necrosis (NEC)/pycnidia (PYC), heading date (Hd) and plant height (Ht)

Trait^a^	IPO323_NEC	IPO323_PYC	IPO323_Hd	IPO88004_NEC	IPO88004_PYC	IPO88004_Hd	IPO92006_NEC	IPO92006_PYC	IPO92006_Hd	IPO86036_NEC	IPO86036_PYC	IPO86036_Hd	St1-03_NEC	St1-03_PYC	St1-03_Hd
IPO323_PYC	**0.81**^**b**^														
IPO323_Hd	**-0.25**	-0.10													
IPO323_Ht	0.02	-0.02	**0.17**												
IPO88004_PYC				**0.82**											
IPO88004_Hd				**-0.49**	**-0.17**										
IPO88004_Ht				0.03	-0.04	**-0.35**									
IPO92006_PYC							**0.71**								
IPO92006_Hd							-0.33	-0.20							
IPO92006_Ht							-0.08	0.05	0.14						
IPO86036_PYC										**0.37**					
IPO86036_Hd										**-0.29**	0.08				
IPO86036_Ht										-0.05	**0.20**	**0.20**			
St1-03_PYC													**0.35**		
St1-03_Hd													**-0.28**	-0.11	
St1-03_Ht													**0.14**	0.13	**0.14**

^a^Trait name consists of the following designations: isolate name IPO323, IPO88004, IPO92006, IPO86036, St1-03 and NEC or PYC, Hd, Ht

^b^values in bold are significant at *p* ≤ 0.05

### Haploblock map and genetic structure

The genotyping of the panel of 217 wheat cultivars on DArTseq revealed 12 690 informative polymorphic DArT-SNP markers which were anchored to the wheat reference genome. Those markers were used for HB construction. A total of 2479 HBs were built within the wheat panel (Table 3). The number of HBs per chromosome spanned from 22 on 4D to 177 on 7A (mean = 112.7). The mean chromosome length coverage was 58.4% and the maximum was for subgenome B (65.9%). The longest HBs were located on subgenome D with a mean of 4.9 Mb. Detailed information is presented in Table 3 and Table S2.

**Table 3:** Detailed information about haploblock map of 217 wheat genotypes based on 12690 DArT-SNP markers

Chromosome	SNPs^a^	HBs^b^	SNPs per HB avg	SNPs per HB max	HB size avg. (Mb^c^)	HB size max (Mb)	HB spacing avg. (Mb)	HB spacing max (Mb)	Chromosome coverage (%)
1A	541	95	4.8	31	4.7	95.4	1.6	24.2	74.5
1B	749	134	4.8	58	3.8	76.4	1.3	10.1	73.9
1D	353	81	3.6	16	4.3	114.6	1.7	17.4	71.1
2A	695	144	4.0	14	3.0	34.3	2.5	69.9	54.6
2B	1 021	163	5.4	21	3.2	47.4	1.7	58.2	65.4
2D	600	131	3.8	12	2.4	50.6	2.6	99.6	48.7
3A	568	123	3.8	22	2.9	42.1	3.2	92.8	47.3
3B	826	164	4.3	19	3.6	78.0	1.5	19.3	71.0
3D	442	91	3.9	23	3.2	33.8	3.6	27.9	47.8
4A	460	84	4.6	22	4.7	86.1	4.2	142.7	52.9
4B	348	77	3.9	13	5.6	94.6	3.1	36.3	64.2
4D	105	22	3.2	9	15.1	161.9	8.3	25.1	65.2
5A	627	135	3.9	22	2.6	71.4	2.7	62.8	49.6
5B	716	137	4.6	14	3.4	46.5	1.9	23.4	64.6
5D	323	76	3.3	9	3.6	37.4	3.7	51.0	47.9
6A	535	100	4.4	16	3.3	54.9	2.9	48.5	54.1
6B	764	134	4.8	24	3.2	24.0	2.2	72.7	58.9
6D	330	74	3.5	31	3.0	37.0	3.5	74.2	46.1
7A	969	177	4.6	15	2.5	35.7	1.7	74.6	59.9
7B	760	139	4.4	17	3.4	83.5	2.0	26.9	63.5
7D	509	105	3.8	18	2.8	56.3	3.3	139.5	45.5
Un	449	93	2.8	7	1.7	13.6	3.5	64.2	33.2

^a^single nucleotide polymorphism

^b^haploblocks

^c^megabase

### Population structure

**Fig. 2:**
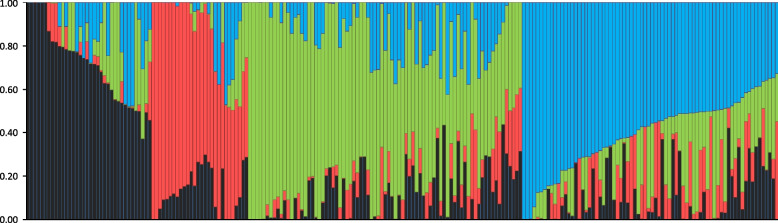
Stacked barplot for 217 wheat genotypes’ population structure based on 2479 haploblocks. Each genotype is represented by a vertical line divided into coloured segments, the lengths and colour of each individual indicate the proportions of the genome attributed to each subpopulation identified through the Structure 2.3.4 program

The Structure analysis indicated four subpopulations which included from 28 (subpopulation 2) to 79 (subpopulation 3) genotypes (Table 4, Fig. 2).

**Table 4:** Distribution of 217 wheat genotypes within four clusters calculated on 2479 haploblocks using structure software

Subpopulation	Wheat genotypes
	Kerubino, Zobel, Famulus, Arktis, Türkis, Rigi, Opal, Schamane, Sokrates, Wiwa, Kranich, Scaro, Pamier, Meteor, Praktik, Agil, Skagen, Butaro, Smaragd, Matix, Kalahari, Garantus, Legenda, Dorota, Akteur, Naridana, Rywalka, Zeppelin, Bamberka, Nelson, Florian, Fregata, Zawisza, Patras, Fakir, Tengri
	Cs Synthetic (6x) 7D, Chinese_Spring, M6 synthetic (W-7984), TE9111, Veranopolis, Israel493, M3 synthetic (W-7976), Courtot, Taichung29, Bulgaria88, Estanzuela Federal, Mazurka, Salamouni, Ch Combin, Begra, MV Lucilla, Renan (RL 248), Edelrun, Réciproc, Valdo, Tadinia, Oceano, Marcopolo, Jaceo, Alhambra, Baletka, Smuga, Ehogold 770/09
	Tulecka, Bockris, Sukces, Tonacja, Zyta, Nutka, Lahertis, Markiza, Bogatka, Figura, Batuta, Jantarka, Henrik, Astoria, Heros, Satyna, KWS Livius, Ostka Strzelecka, Kohelia, Platin, Arkadia, Glaucus, Estivus, Ludwig, Pengar, Eron, Fermi, Lavantus, Matheo, Capone, Tobak, Manager, Gordian, Desamo, Elixer, Mulan, Look, Pionier, Terroir, Solitar, Magnus, Bombus, Operetka, Dekan, Bystra, Sailor, Rumor, Kobiera, Askalon, Forkida, Bagou, Lear, Kredo, Joker, Memory, Belenus, Natula, Meister, Turnia, Primus, Julius, Ostroga, Diskus, Artagnan, Belepi, Addict, Etana, Florett, KWS Dacanto, Forum, Speedway, Fidelius, Mandub, Wydma, Arina, Mikula, Mewa, Muza, Liwilla
	Kampana, Muszelka, Alcazar, Kris, Rapsodia, Lithium, Waxy, Kepler, Olivin, Ionesco, Fructidor, Winnetou, Chilton, Samurai, Linus, Intro, RGT Frenezio, Celebration, Atomic, Barok, Sophytra, RGT Ampiezzo, Alchemy, Tuareg, Oxal, Mentor, Boomer, Gabrio, Xantippe, RGT Kilimanjaro, Avalon (W 2564), Pueblo, Mandragor, Jenga, Eperon, Diamento, Starway, Banderola, Arezzo, Solognac, Colonia, Tentation, Frument, Edgar, Diderot, KWS Ozon, Elipsa, Caroll, Kantao, Zappa, Tabasco, Apache, Balance, Nocibe, Calcio, KWS Erasmus, Evolution, Thalys, Zephyr, Syllon, Avenir, Granamax, Torrild, Riband, Amifor, Flame, Grapeli, Dacanto, Salutos, Descartes, Artist, KWS Magic, Armada, RGT Djoko

**Fig. 3:**
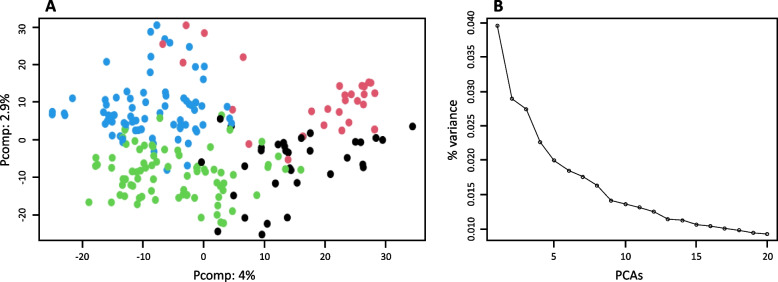
Principal component analysis on the 2479 haploblocks of 217 wheat genotypes. **A** Scatterplot of the 1st and 2nd components. Each cultivar is represented by coloured dots corresponding to the four clusters identified in Structure analysis. **B** Scree plot of the percentage of the variance explained by the first 20 PCs

All the subpopulations showed a relatively high degree of admixture, which ranged from 60% of genotypes with less than 80% of proportions of the genome attributed to the assigned subpopulation (group 2) to 85% (group 4) (Table S3). The principal component analysis (PCA) revealed a mild effect within the wheat panel. The first two principal components (PCs) explained about 7% of the genetic variance (Fig. 3). Separation within subpopulations was not clear and clusters of genotypes were linked to each other (Fig. 3A). As long as PC analysis corrects for spurious associations at a global level of genetic variation, the GWAS methodology utilized in our study incorporated a population structure effect based on PCA. According to the analysis of the variance explained by the first 20 PCs (Fig. 3B), the first three PCs accounted for the population structure effect in GWAS.

As for the cultivars’ origin, there was no significant relationship between groups (subpopulations) and their origin. Subpopulation 1 (36 genotypes) consisted of cultivars originating from European countries, mainly from Germany but also from Poland, France, Switzerland and others. The least numerous subpopulation, i.e. no. 2 (28 genotypes), comprised cultivars from France, other European countries, China, USA and Japan. It was noted that subpopulation 3 (79 genotypes) consisted of cultivars from Germany, Poland, France and other European countries, whereas subpopulation 4 (74 genotypes) included mainly French and German wheat genotypes (detailed information in Table S3). Subpopulation 2 contains the most varieties with identified STB resistance genes (13 out of 22 genotypes) used in this study as sources of known resistance (with different origins); four were grouped in subpopulation 3 and five genotypes with known resistance genes were assigned to subpopulation 4. The average value of NEC in subpopulations ranged from 40% (in group 3) to 57% (in group 2). In the case of PYC average values ranged from 14% (groups 3 and 4) to 22% (group 2) (data not shown).

### GWAS analysis

**Fig. 4:**
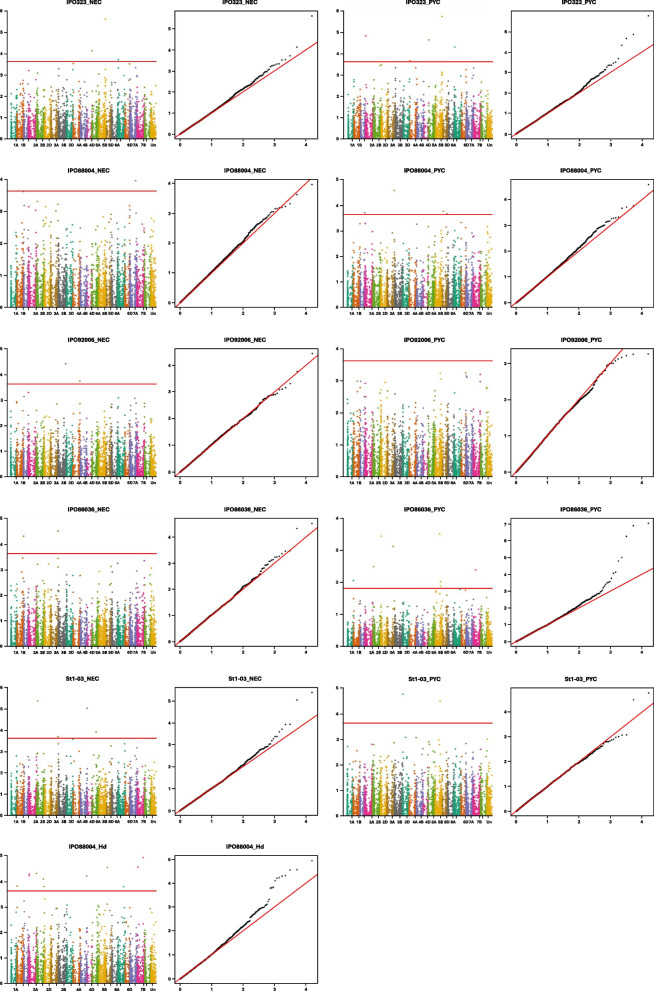
Manhattan and Q-Q plots after GWAS for disease parameters (NEC, PYC and Hd) after treatment with five *Z. tritici* isolates using217 wheat genotypes. The red line indicated a threshold significance level of 5% corresponding to a–log10(*p*) > 3.64

**Fig. 5:**
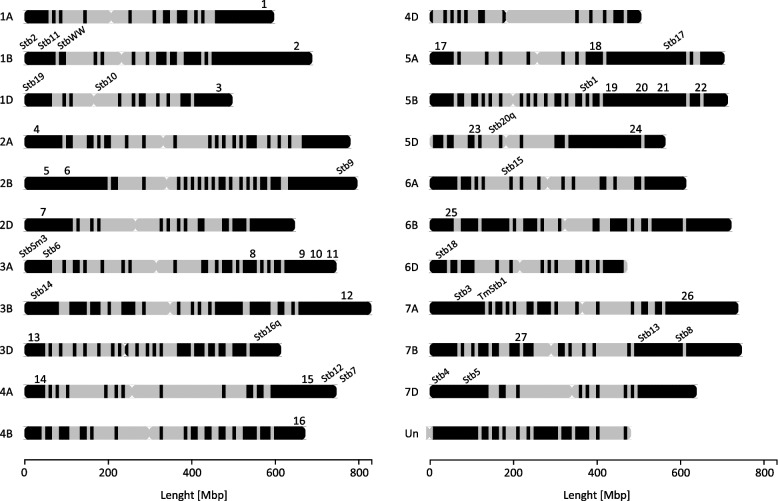
Simplified map of wheat chromosomes. Black areas represent the positions of all the haploblocks (HBs) that have been identified based on the distribution of 12,690 DArT-SNP markers within a panel of 217 wheat genotypes. Numbers 1–27 indicate the positions of HBs significantly associated with resistance to *Z. tritici* (listed in Table 5). Additionally, the locations of major *Stb* genes are indicated based on Brown et al. [18–20, 38]. Detailed information about the HBs is included in Table S2

The GWAS analysis of disease symptoms caused by five STB isolates and disease escape traits (Hd and Ht) revealed 27 HBs (and corresponding haplotypes, HTs) significantly associated with analysed traits due to the *p*-value being above the genome-wide significance threshold line (Table 5, Fig. 4, Table S4). The identified HBs were dispatched over the wheat genome (Fig. 5).

**Table 5:** Haploblocks associated with Septoria tritici disease parameters within 217 wheat genotypes according to haploblock-based GWAS analysis

**No.**^a^	**HB**^**b**^	**HT**^**c**^	**Trait**^**d**^	**Location**	***p*-value**	**R**^**2**^	**Freq.**^**e**^
1	Chr1A_HB86	_HT9	IPO86036_PYC	Chr1A:577314364–577370857	7.36961 × 10^−5^	7.9%	15
2	Chr1B_HB123	_HT1	IPO86036_NEC	Chr1B:668628620–669101252	4.66959 × 10^−5^	11.4%	203
3	Chr1D_HB68	_HT4	IPO88004_PYC	Chr1D:464629693–464864436	1.92482 × 10^−4^	7.7%	20
4	Chr2A_HB11	_HT23	IPO323_PYC	Chr2A:15753608–16016953	1.39237 × 10^−5^	13.2%	45
5	Chr2B_HB25	_HT3	IPO86036_PYC	Chr2B:43237442–45349362	9.69905 × 10^−6^	12.0%	21
6	Chr2B_HB41	_HT1	St1-03_NEC	Chr2B:100325164–103893978	4.03511 × 10^−6^	6.0%	12
7	Chr2D_HB31	_HT3	IPO86036_PYC	Chr2D:27085457–29281784	1.24126 × 10^−7^	19.7%	11
8	Chr3A_HB68	_HT1	IPO86036_PYC	Chr3A:556548390–565574116	5.33099 × 10^−7^	19.9%	13
9	Chr3A_HB93	_HT4	St1-03_NEC	Chr3A:697456447–700757762	1.93480 × 10^−4^	7.1%	13
10	**Chr3A_HB98**	**_HT2**	IPO86036_NEC	Chr3A:710602814–710771071	2.98085 × 10^−5^	10.7%	170
11	Chr3A_HB100	_HT2	IPO88004_PYC	Chr3A:713210829–714298386	2.57228 × 10^−5^	12.0%	55
12	Chr3B_HB123	_HT3	IPO92006_NEC	Chr3B:736134234–737117265	3.67125 × 10^−5^	7.3%	18
13	Chr3D_HB11	_HT4	St1-03_PYC	Chr3D:5760912–6141995	1.68309 × 10^−5^	7.4%	47
14	Chr4A_HB10	_HT3	IPO323_PYC	Chr4A:29387604–29625559	2.09959 × 10^−4^	5.2%	11
15	Chr4A_HB74	_HT2	IPO92006_NEC	Chr4A:708958917–710176236	1.72792 × 10^−4^	6.6%	20
16	Chr4B_HB68	_HT1	St1-03_NEC	Chr4B:652962151–653446105	4.03511 × 10^−6^	3.9%	200
17	Chr5A_HB4	_HT2	IPO323_NEC	Chr5A:6894902–7493176	7.22619 × 10^−5^	4.6%	30
			IPO323_PYC		2.17891 × 10^−5^	6.1%	30
18	Chr5A_HB29	_HT1	St1-03_NEC	Chr5A:382294092–382769155	6.18852 × 10^−1^	2.1%	182
		_HT6	St1-03_NEC	Chr5A:382294092–382769155	1.16041 × 10^−4^	1.8%	27
19	**Chr5B_HB47**	**_HT1**	IPO86036_PYC	Chr5B:422408166–422654695	8.92333 × 10^−8^	21.5%	206
20	Chr5B_HB69	_HT3	IPO86036_PYC	Chr5B:502016122–513647963	8.51117 × 10^−5^	11.5%	12
			St1-03_PYC		3.16365 × 10^−5^	8.8%	12
21	Chr5B_HB71	_HT18	IPO86036_PYC	Chr5B:519034445–526607377	1.65501 × 10^−4^	12.3%	29
22	Chr5B_HB118	_HT2	IPO323_NEC	Chr5B:641314832–642840347	2.35844 × 10^−6^	12.2%	44
			IPO323_PYC		1.70341 × 10^−6^	8.3%	44
23	Chr5D_HB10	_HT1	IPO88004_PYC	Chr5D:105062991–108688443	1.70060 × 10^−4^	0.2%	179
		_HT3	IPO88004_Hd		2.70031 × 10^−5^	11.2%	11
24	Chr5D_HB51	_HT5	IPO88004_PYC	Chr5D:491095719–493912844	2.12416 × 10^−4^	3.2%	188
25	**Chr6B_HB31**	**_HT2**	IPO323_NEC	Chr6B:39302769–40061097	1.87952 × 10^−4^	6.7%	41
			IPO323_PYC		4.73922 × 10^−5^	8.2%	41
26	Chr7A_HB107	_HT3	IPO88004_NEC	Chr7A:606257632–607536273	1.08096 × 10^−4^	0.8%	36
27	**Chr7B_HB36**	**_HT1**	IPO86036_PYC	Chr7B:203001232–212554489	1.59239 × 10^−5^	11.2%	195

^a^number ID indicated on simplified map of wheat (Fig. 5)

^b^haploblock

^c^haplotype

^d^trait name consists of the following designations: isolate name IPO323, IPO88004, IPO92006, IPO86036, St1-03 and NEC (percentage of leaf area covered by necrosis), PYC (percentage of leaf area covered by pycnidia) or Hd (heading date)

^e^haplotype frequency in population; HB and HT in bold indicate sequence variants that decrease disease symptoms (NEC and/or PYC)

From 21 wheat chromosomes significant HB were not found in four chromosomes (Chr4D, Chr6A, Chr6D and Chr7D. Most identified HBs were significantly associated with a single trait, and five were associated with two traits (PYC and NEC) for a single isolate: Chr5A_HB4, Chr5B_HB118, Chr6B_HB31 The HB; Chr5B_HB69, was significantly associated with PYC trait triggered by two isolates (IPO86036 and St1-03). The HB; Chr5D_HB10 was significantly associated with two traits (PYC and Hd) of the isolate IPO88004. The explained phenotypic variation of the significant HBs ranged from 0.2% to 21.5% (Table 5).

The HBs had a mean value of explained phenotypic variation of 8.7% and median of 7.8%. Three HBs had explained phenotypic variation above 19%: Chr5B_HB47, Chr3A_HB68 and Chr2D_HB31. All of them were significantly associated with the PYC trait of the IPO86036 isolate. The GWAS analysis of heading date and plant height revealed 11 HBs. Only one of them (Chr5D_HB10) was also associated with the resistance trait (Table 5). The frequency of individual HTs varied from 11 to 206 (Table 5). Detailed information about the cultivars possessing each HT from significant HB is included in Table S5.

**Fig. 6:**
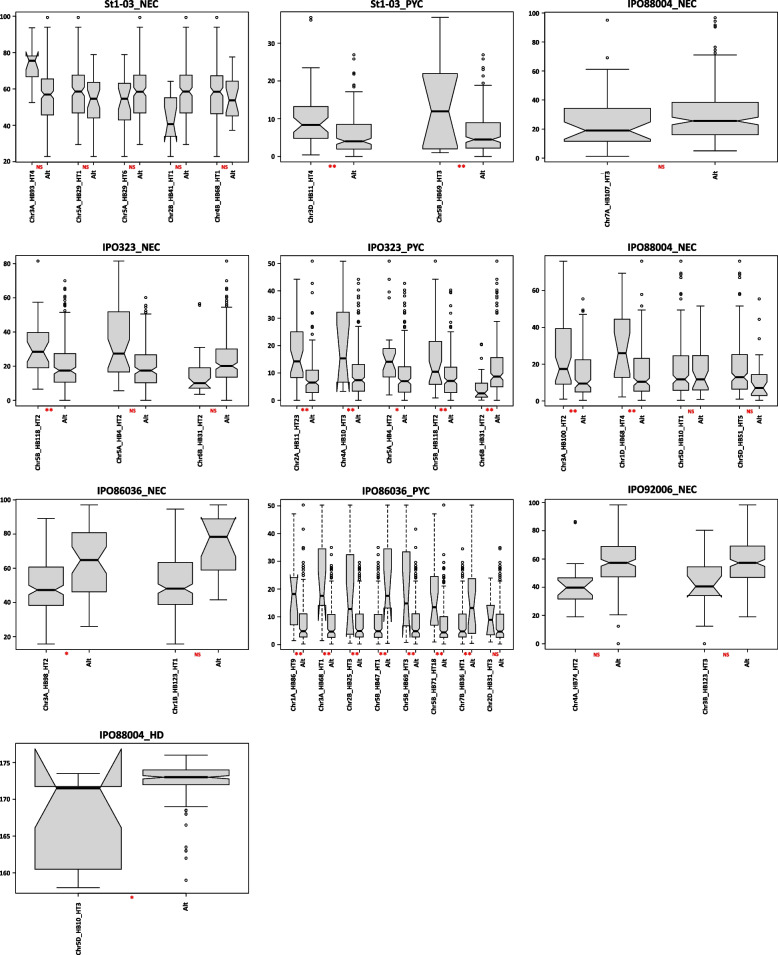
Box plots for STB disease parameters (NEC, PYC; the value on the axis is expressed as a percentage) and escape trait (Hd; the value on the axis is expressed as number of days) made on the basis of haplotypes; each picture represents a different phenotypic trait; Alt – means the remaining haplotypes from the haploblock of interest, NS – not significant, *- significant at *P*-val < 0.05, **- significant at *P*-val < 0.01

Box plots for HT phenotypic values of the investigated traits based on Fisher’s test statistic are shown in the Fig. 6 and the impact of significant HTs for each isolate was compared. Chr3A_HB98_HT2 significantly (*p*-value < 0.05) differed from other HTs and showed a positive influence on the trait IPO86036_NEC decreasing level of necrotic area. In the case of IPO86036_PYC two HTs were identified (Chr5B_HB47_HT1 and Chr7B_HB36_HT1) that showed statistically significant (*p*-value < 0.01) differences from the other HTs decreasing the level of the investigated trait. Apart from that only one HT (Chr6B_HB31_HT2) was statistically significant (*p*-value < 0.01) and decreased the trait IPO323_PYC; all remaining HTs did not show significant differences or significantly increased disease parameters. In the case of the Chr5D_HB10_HT3 of the Hd trait the differences between this haplotype and others were statistically significant (*p*-value < 0.01) and reduced the trait value (Fig. 6).

## Discussion

The study utilized a diverse collection of *Z. tritici* isolates [39], revealing a wide spectrum of virulence. The different frequency distributions observed for both disease parameters in the studied wheat cultivars indicate a diverse resistance response, predominantly characterized by high broad-sense heritability. Additionally, a relatively strong positive correlation between necrosis and pycnidia coverage was observed, consistent with reports from some authors [23, 40], although certain studies suggest separate genetic control over leaf necrosis and pycnidia coverage [41, 42].

To date, 23 major STB resistance genes and over 160 QTL have been identified and mapped using bi-parental populations or association mapping panels [19, 20, 23, 34]. Recently, haplotype-based GWAS became a powerful approach, as haploblocks, being groups of neighbouring SNPs that are inherited together, offer higher resolution in association mapping compared to individual SNP markers [29, 43, 44]. By analysing combinations of SNPs instead of single variants, haploblocks enhance statistical power and precision in identifying genome regions associated with traits of interest. In plant studies, where recombination is limited in certain genome regions, the use of haploblocks can better reflect actual inheritance and genetic interactions, leading to more accurate association mapping results [29, 43, 44]. This approach was used by Yates et al. [21], who identified 26 chromosomal intervals associated with four STB resistance traits. They found that most of that region overlapped with known STB resistance loci. Similarly, our haplotype-based GWAS analysis revealed 27 genetic regions significantly associated with resistance traits. These regions were distributed within almost all wheat chromosomes. A few of them were located on the same chromosome but were genetically distant from each other (Fig. 5). In contrast, the regions described by Yates et al. [21] were distributed across 13 chromosomes and individual intervals explained from 1.9% to 10.6% of the phenotyping variance for each trait. The identified chromosomal regions of wheat statistically significantly explained the phenotypic variance ranging from 0.2% to 21.5%. Among these, those with the highest values may be valuable in breeding for resistance to STB.

Among the statistically significant haplotypes identified, four of them were of particular interest, as they contribute to the reduction of disease symptoms. One of the identified significant HBs on the long arm of chromosome 3A turned out to be of particular interest. The Chr3A_HB98 associated with leaf necrosis coverage by isolate IPO86036 showed at the same time a statistically significant decrease in the extent of necrotic area which led to limitation of pycnidia formation area. This suggests that we are dealing with a valuable source of resistance, especially considering wheat cultivars possessing the described HT (Table S5). Hitherto, chromosome 3A has been often described in the literature to harbour *Stb6* and *StbSm3* genes [18]. Radecka-Janusik and Czembor [40] described in this chromosome a QTL for the adult plant and seedling resistance after inoculation with the IPO323 isolate. However, the locus is located on the short arm of the chromosome and is very distinct from our HB (Fig. 5), suggesting identification of a new region of resistance with 10.7% of explained phenotypic variance.

The significant HB on chromosome 5BL at a close distance (about 4Mb) to the major gene *Stb1* was also one of the HBs affecting the reduction of the disease trait, mainly leaf coverage by pycnidia. Yates et al. [21] used GWAS to identify and describe an interval on chromosome 5B that was at a distance of 20 Mb from the one identified in our study. In the approximate region Piaskowska et al. [23] mapped STB *QStb.ihar-5B* QTL which explaining 11.9% of the phenotypic variation at the adult plant stage. Chr5B_HB47 explained greater phenotypic variance (21.5%), which may have better utilization potential in breeding programmes.

Yates et al. [21] after a natural STB infection detected genetic intervals on chromosome 6B associated with pycnidia prevalence (namely pycnidia density within lesions). Three of them were clustered on the long arm, while Chr6B_HB31 from the current study was mapped on the short arm of chromosome 6B and was associated with IPO323 necrosis as well as pycnidia coverage. Previous reports described QTL resistance to IPO323 in the centromeric region of chromosome 6B and others associated with resistance to different isolates [18]. None of these QTL co-localized with Chr6B_HB31, which may suggest a new resistance region.

To date two major *Stb* genes have been mapped on chromosome 7BL [18]. Also, several STB associated QTL on chromosome 7B have been described in the literature [21, 25, 45]. For example, *QStb.risø-7B* explained 12.2% of phenotypic variation in pycnidia coverage in response to a mixture of 11 *Z. tritici* isolates [45]. The current study showed the Chr7B_HB36 region to be significantly associated with STB resistance and a decreased percentage of leaf coverage after inoculation by the IPO86036 isolate (11.2% of explained variance). Because the localization was at 62 Mb distance from that previously reported [21], it is possible that Chr7B_HB36 region correspond a novel region in chromosome 7B responsible for STB resistance. Other GWAS-based studies allowed QTL to be identified on nine chromosomes that exerted a minor to modest effect on the trait [25]. Among them was a minor QTL on chromosome 7BL but noteworthy significant HB, which exhibited improved STB resistance, and the authors suggested that it could be the *Stb8* gene [25].

For the purposes of comparative analysis of identified MTAs, a set of genotypes containing previously identified major Stb resistance genes was also used in the study. None of these genes were detected in the differential panel or in other wheat varieties, despite inoculation with IPO323 and IPO88004 – isolates that were previously used to identify genes *Stb6*, *Stb15* and *Stb18*. There could be several reasons for this situation, among them genetic background and environmental interaction, which can significantly modify the expression of resistance genes. *Stb6* seems to be a suitable example, as for the 13 lines/cultivars of the differential panel presumed to contain this gene, we noted high variability in both disease parameters after IPO323 inoculation, with NEC values ranging from 3.5% to 55.6% and PYC values ranging from 0% to 44.2%. On the other hand, the absence of *Stb15* detection may be related to the plant growth stage at which the experiments were conducted, as it is known to confer resistance in seedlings rather than adult plants [36, 46]. An association between the *Stb15* region and pycnidia density within lesions trait (in adult plants) has been reported [21], however, the same study did not detect an association of this region with the percentage of leaf area covered by necrotic lesions in adult plants, which was the trait that we were also analyzing.

The previously described major *Stb* genes in literature do not confer complete resistance at the adult stage, but rather have a quantitative nature with medium and large effects, which might not always be detected at reduced GWAS resolution. If the LD decays rapidly in the studied population, the associated regions might not directly overlap with known genes due to the fine-scale resolution of GWAS [47]. The statistical power of GWAS to detect associations with major genes can be affected by the size of the effect, allele frequency, and the sample size. Major genes with a strong effect but low allele frequency as well as genes with small effect but high allele frequency might require a larger sample size to be detected [29]. Therefore, *Stb18* may have been overlooked due to the adopted statistical methods, given that it is a gene with rather small effect. It was detected inconsistently in a biparental population inoculated with IPO323, explaining up to 12.7% of the variation in percentage of leaf area bearing pycnidia [42]. Considering the advantage of biparental populations in detecting of minor genes, it is unsurprising that it was not identified in our study. Regarding the allele frequency, as far as we know, there has been no study to date investigating the prevalence of *Stb18* in wheat cultivars worldwide.

Apart from the minimum allele frequency requirement, the chance of detecting major *Stb* genes may also have been reduced by imperfections in the phenotypic data. Firstly, we should consider the prevailing weather conditions. As we conducted the experiments on adult plants with flag leaves fully emerged (inoculation at the beginning of June and assessment at the turn of June and July, depending on the year of the experiment), the ambient temperatures during the day may have exceeded 30°C and even risen to 50°C inside the polytunnels. This may have caused rapid maturing of the plants as well as disrupted disease development. Secondly, the wheat panel used in our study displayed rather high diversity in terms of noted heading dates (the difference between the earliest and latest genotypes was approximately 14 days across all experiments). Therefore, during the experiment, some plants may have already begun to dry up (especially with high temperatures occurring), making it harder to distinguish between necrosis caused by maturing and necrosis caused by the disease. This would not be an issue in experiments on seedlings, as we can treat the seeds and seedlings to be closer in development at the time of inoculation.

The EnsemblPlants database [48] was searched for the four genetic regions (Chr3A_HB98 – 168 kbp region on chromosome 3A; Chr5B_HB47 – 247 kbp, 5B; Chr6B_HB31 – 758 kbp, 6B; and Chr7B_HB36 – 9553 kbp, 7B) identified in our study that statistically significantly contributed to the decrease of phenotypic value of the disease parameters (Table 5). According to this information, 54 genes located inside these regions coded different proteins or protein domains. Detailed information is included in the Supplementary Table S6. Among them, the most closely related to the known genes involved in the plants’ resistance response to pathogens were a few genes on chromosome 6B that encode leucine-rich repeat domains (TraesCS6B02G059900, TraesCS6B02G060000, TraesCS6B02G060100) and protein kinase domain-containing domain (TraesCS6B02G059800) as well as NAD(P)-binding domain-containing protein (TraesCS6B02G060200). The HB located on 3A covered encode LRR domain (TraesCS3A02G478500). This may support the hypothesis that the identified genetic region Chr6B_HB31 on 6BS carries a potential region for pathogen resistance, which in our study contributed to resistance to *Z. tritici* isolate IPO323.

It is a well-established that taller plants and those with a later heading date reduce the spread of STB spores to the upper leaves. This limits the likelihood of contact between the pathogen and the host [36, 40, 46]. QTL for height and heading date were identified simultaneously in adult plant resistance experiments [23, 40, 49]. GWAS analysis in our study revealed one HT associated with the Hd trait in on the short arm of chromosome 5D (Chr5D_HB10_HT3). Chr5D_HB10_HT3 was detected in the same region as the IPO88004_PYC trait and it may affect the resistance as weak but significant negative correlation was observed. In our case this chromosome region was associated with the resistance trait as box plot visualization revealed that Chr5D_HB10_HT3 is associated with earlier heading date when compared to the rest of HTs from this HB. In fact, there is a heading date-associated region on chromosome 5D. Yoshida et al. [50] described the *Vrn-D4* centromeric region and confirmed that the *Vrn-D4* effect on flowering time is modulated by vernalization.

The correlations between escape traits and the disease parameters measured in our study were mostly weak or insignificant, as the inoculation was performed when the flag leaves were fully emerged (not before the 39 BBCH stage), and disease assessment was conducted on these leaves as well. Consequently, we did not anticipate the escape traits to be markedly evident in the GWAS. Studies that have identified genes for escape traits typically rely on natural infection or artificial inoculation but at earlier stages of plant growth, such as the two-leaf seedling stage or the beginning of tillering [48, 51, 52].

*Stb* resistance gene/QTL interactions may result in a more complex plant resistance response. Tabib Ghaffary et al. [42] observed an epistatic effect of 3AS QTL with6DS QTL, which conducted to a lower level of pycnidia coverage, but it was not statistically significant. At the same time, the authors described the additive effect of 6DS and 7D QTL. This proves that the role of each described resistance gene is not always direct and clear. In our investigation, there are novel genomic regions that need to be explored. In most cases the most resistant genotypes possessed four significant HTs (Table 5, Table S5), conducting a high decrease in disease parameters. This information could be valuable for breeders because breeding for resistance remains the most economical, effective, and environmentally friendly strategy to prevent STB.

## Conclusions

GWAS analysis allowed us to distinguish significant chromosomal regions with a positive effect on STB resistance. Four significant genetic regions – Chr3A_HB98, Chr5B_HB47, Chr6B_HB31 and Chr7B_HB36 – can potentially be considered as new sources of resistance to be utilized in wheat breeding programs. Conversely, larger haploblocks might encompass coding regions associated with the trait of interest. The list of valuable varieties containing the identified HBs wheat provides promising material for further molecular analysis of resistance loci and development of breeding programmes.

## Materials and methods

### Plant and fungal material

In this study we used a panel of 217 wheat genotypes comprising 83 winter cultivars listed on the national descriptive list COBORU as well as 110 winter cultivars from other countries – mainly from Europe, 22 cultivars/lines that are known to carry STB resistance loci and 2 susceptible checks (Table S7, Table S8). The inclusion of varieties/lines possessing known *Stb* resistance genes (differential panel) in the analysis aimed to confirm or exclude their presence in the tested wheat variety panel. The panel was evaluated at the adult plant stage.

Five *Z. tritici* isolates of diverse pathogenicity were chosen for the pathology tests: IPO323, IPO88004, IPO92004, IPO86036 and St1-03 [39]. The isolates were grown on Petri dishes containing YMA medium consisting of 4 g of yeast, 4 g of maltose, 4 g of sucrose and 30 g of agar per 1 L of water [5]. The dishes were kept in the dark at a constant 20°C temperature. The spores were collected after 3 days and stored at -80°C. Before the inoculation, the concentration of the spore suspension was adjusted to 10–15 × 10^6^ spores/mL and a few drops of a surfactant (TWEEN 20, Sigma-Aldrich) were added.

### STB resistance tests

The wheat panel was tested at the adult plant stage under polytunnel conditions in the years 2015–2019. To prevent contamination, one isolate was tested per season. For each test, the seeds were sown in two randomized blocks, in 1-m-long rows spaced at 18 cm. Pycnidiospore suspension (100 mL/1 m2) spray was applied when all genotypes developed flag leaf. The inoculation took place in the evening to help retain the moisture on the leaf surface overnight and therefore promote infection. To maintain relatively high humidity during the tests, polytunnels were equipped with a sprinkler irrigation system providing water three times a day. Assessment of disease development took place when approximately 80% of susceptible check Begra’s flag leaves surface became necrotic (generally 21 days after inoculation). At least five flag leaves were sampled per replicate. In each test, two disease parameters were evaluated: the percentage of leaf area covered by necrotic lesions (NEC) and the percentage of leaf area covered by lesions bearing pycnidia (PYC) according to Piaskowska et al. [23]. The pictures of the leaves were taken with Canon EOS 5D Mark II camera equipped with CANON COMPACT-MACRO EF 50 mm 1:2.5 lens. Resolution of the acquired pictures was 5616 × 3744 pixels. Disease escape parameters – heading date (Hd) and plant height (Ht) – were also measured. The analysis of variance (ANOVA) was performed with XLSTAT software (Addinsoft, version 2016.02.28540) and broad-sense heritability (*h*^*2*^) was calculated for both disease parameters [53]. Furthermore, to estimate the relationship between necrosis/pycnidia coverage, plant height (cm) and heading date (days) (calculated from 1 January), Pearson’s correlation coefficient was calculated.

### Genotyping and haploblock construction

The 217 wheat cultivars were genotyped on the DArTseq platform by Diversity Arrays Technology, Pty. Ltd., Australia [54]. For further analysis SNP markers with the genomic location previously designated by the DArT platform according to the Wheat_ChineseSpring04 reference model were employed. Missing sequence data were imputed using A.mat R function [55] using an expectation maximization algorithm based on the multivariate normal distribution. Further analysis was conducted using haploblocks identified by Haploview 4.2 [56], based on the solid spine of linkage disequilibrium and the extended spine if D’ > 0.8. The resulting haploblocks (HB), which are sequences of genetic markers and combinations of SNP variants within HBs called haplotypes (HT), were transformed into a 0/1 matrix format using the Haploview2gapit Python script [31]. The visualisation of obtained HBs was prepared in chromoMap R script [57]. Haplotypes with minor allele frequency (MAF) < 5% were removed from further analysis. Visualization in the box plots of significant HTs in HBs was prepared with the R package ‘graphics’ version 4.2.1 according to Chambers’ [58] instructions: when a notch is drawn on each side of the boxes and when the notches of two plots do not overlap, it is strong evidence that the two medians differ. A comparison of the variance of the data sets was performed using Fisher’s test.

### Population structure and GWAS analysis

The population structure (Q value) was designated in Structure 2.3.4 [59] with an admixture model without using prior information. The optimal value of K ranges from 1 to 10 with ten independent rounds. The analysis was performed with a burn-in period of 10 000 and Markov Chain Monte Carlo (MCMC) with 10 000 repetitions. The PCA analysis and kinship similarity matrix preparation was conducted with PCA and K.mat R functions respectively. The GWAS analysis was prepared separately for each phenotype data set with the GWAS function from the rrBLUP R package [55, 60] with population structure effects and similarity matrix employment. The Manhattan and QQ plots were visualised in the qqman R package [61]. The genome-wide significance level of 5%, which was –log10(*p*) > 3.64 [62], was the threshold for significant HB trait associations. The explanation of phenotypic variance (R^2^) was calculated for significant HBs in the CJAMP R package [63].

### Supplementary Information

Supplementary Material 1.

Supplementary Material 2.

Supplementary Material 3.

Supplementary Material 4.

Supplementary Material 5.

## Data Availability

Data generated or analysed during this study are included in the main text article and its supplementary files. Data not included are available from the correspondent author on reasonable request.

## References

[1] FAOSTAT. Food balance, new food balances. http://www.fao.org/faostat/en/#data/FBS. Accessed Oct 2023.

[2] FAOSTAT. Production, crops. http://www.fao.org/faostat/en/#data/QC. Accessed Oct 2023.

[3] Ramankutty N, Mehrabi Z, Waha K, Jarvis L, Kremen C, Herrero M, Rieseberg LH (2018). Trends in global agricultural land use: implications for environmental health and food security. Annu Rev Plant Biol.

[4] Orton ES, Deller S, Brown JK (2011). *Mycosphaerella graminicola*: from genomics to disease control. Mol Plant Pathol.

[5] Eyal Z, Scharen AL, Prescott JM, van Ginkel M (1987). The septoria diseases of wheat: concepts and methods of disease management.

[6] McDonald BA, Linde C (2002). Pathogen population genetics, evolutionary potential, and durable resistance. Annu Rev Phytopathol.

[7] Dean R, Van Kan JA, Pretorius ZA, Hammond-Kosack KE, Di Pietro A, Spanu PD, Rudd JJ, Dickman M, Kahmann R, Ellis J, Foster GD (2012). The Top 10 fungal pathogens in molecular plant pathology. Mol Plant Pathol.

[8] Savary S, Willocquet L, Pethybridge SJ, Esker P, McRoberts N, Nelson A (2019). The global burden of pathogens and pests on major food crops. Nat Ecol Evol.

[9] Zhang CJ, Chen GX, Gao XX, Chu CJ (2006). Photosynthetic decline in flag leaves of two field-grown spring wheat cultivars with different senescence properties. S Afr J Bot.

[10] Fones H, Gurr S (2015). The impact of Septoria tritici Blotch disease on wheat: an EU perspective. Fungal Genet Biol.

[11] Fraaije BA, Cools HJ, Fountaine J, Lovell DJ, Motteram J, West JS, Lucas JA (2005). Role of ascospores in further spread of QoI-resistant cytochrome b alleles (G143A) in field populations of *Mycosphaerella graminicola*. Phytopathology.

[12] Torriani SFF, Brunner PC, McDonald BA, Sierotzki H (2008). QoI resistance emerged independently at least 4 times in European populations of *Mycosphaerella graminicola*. Pest Manag Sci.

[13] Cools HJ, Mullins JGL, Fraaije BA, Parker JE, Kelly DE, Lucas JA, Kelly SL (2011). Impact of recently emerged Sterol 14α-Demethylase (CYP51) variants of *Mycosphaerella graminicola* on azole fungicide sensitivity. Appl Environ Microb.

[14] Cools HJ, Fraaije BA (2013). Update on mechanisms of azole resistance in *Mycosphaerella graminicola* and implications for future control. Pest Manag Sci.

[15] Heick TM, Matzen N, Jørgensen LN (2020). Reduced field efficacy and sensitivity of demethylation inhibitors in the Danish and Swedish *Zymoseptoria tritici* populations. Eur J Plant Pathol.

[16] Lucas JA, Hawkins NJ, Fraaije BA. The evolution of fungicide resistance. Adv Appl Microbiol. 2015;90:29–92. 10.1016/bs.aambs.2014.09.001 25596029

[17] Birr T, Hasler M, Verreet J-A, Klink H (2021). Temporal changes in sensitivity of *Zymoseptoria tritici* field populations to different fungicidal modes of action. Agriculture.

[18] Brown JKM, Chartrain L, Lasserre-Zuber P, Saintenac C (2015). Genetics of resistance to Zymoseptoria tritici and applications to wheat breeding. Fungal Genet Biol.

[19] Yang N, McDonald MC, Solomon PS, Milgate AW (2018). Genetic mapping of *Stb19*, a new resistance gene to *Zymoseptoria tritici* in wheat. Theor Appl Genet.

[20] Langlands-Perry C, Cuenin M, Bergez C, Krima SB, Gélisse S, Sourdille P, Valade R, Marcel TC (2022). Resistance of the wheat cultivar ‘Renan’ to septoria leaf blotch explained by a combination of strain specific and strain non-specific QTL mapped on an ultra-dense genetic map. Genes.

[21] Yates S, Mikaberidze A, Krattinger SG, Abrouk M, Hund A, Yu K, Studer B, Fouche S, Meile L, Pereira D, Karisto P, McDonald BA (2019). Precision phenotyping reveals novel loci for quantitative resistance to septoria tritici blotch. Plant Phenomics.

[22] Dutta A, Croll D, McDonald BA, Krattinger SG (2021). Genome-wide association study for septoria tritici blotch resistance reveals the occurrence and distribution of *Stb6* in a historic Swiss landrace collection. Euphytica.

[23] Piaskowska D, Piechota U, Radecka-Janusik M, Czembor P (2021). QTL mapping of seedling and adult plant resistance to septoria tritici blotch in winter wheat cv. Mandub (*Triticum aestivum* L.). Agronomy.

[24] Tabib Ghaffary SM, Chawade A, Singh PK (2018). Practical breeding strategies to improve resistance to Septoria tritici blotch of wheat. Euphytica.

[25] Muqaddasi QH, Zhao Y, Rodemann B, Plieske J, Ganal MW, Röder MS (2019). Genome-wide association mapping and prediction of adult stage Septoria tritici blotch infection in European winter wheat via high-density marker arrays. Plant Genome.

[26] Kollers S, Rodemann B, Ling J, Korzun V, Ebmeyer E, Argillier O, Hinze M, Plieske J, Kulosa D, Ganal MW, Röder MS (2013). Genetic architecture of resistance to Septoria tritici blotch (*Mycosphaerella graminicola*) in European winter wheat. Mol Breeding.

[27] Miedaner T, Zhao Y, Gowda M, Longin CFH, Korzun V, Ebmeyer E, Kazman E, Reif J (2013). Genetic architecture of resistance to Septoria tritici blotch in European wheat. BMC Genomics.

[28] Gurung S, Mamidi S, Bonman JM, Xiong M, Brown-Guedira G, Adhikari TB (2014). Genome-wide association study reveals novel quantitative trait loci associated with resistance to multiple leaf spot diseases of spring wheat. PLoS ONE.

[29] Korte A, Farlow A (2013). The advantages and limitations of trait analysis with GWAS: a review. Plant Methods.

[30] Qian L, Hickey LT, Stahl A, Werner CR, Hayes B, Snowdon RJ, Voss-Fels KP (2017). Exploring and harnessing haplotype diversity to improve yield stability in crops. Front Plant Sci.

[31] Luján Basile SM, Ramírez IA, Crescente JM, Conde MB, Demichelis M, Abbate P, Rogers WJ, Pontaroli AC, Helguera M, Vanzetti LS (2019). Haplotype block analysis of an Argentinean hexaploid wheat collection and GWAS for yield components and adaptation. BMC Plant Biol.

[32] Tabib Ghaffary SM, Faris JD, Friesen TL, Visser RGF, van der Lee TAJ, Robert O, Kema GHJ (2012). New broad-spectrum resistance to Septoria tritici blotch derived from synthetic hexaploid wheat. Theor Appl Genet.

[33] Alemu A, Brazauskas G, Gaikpa D, Henriksson T, Islamov B, Jørgensen LN, Koppel M, Koppel R, Liatukas Ž, Svensson JT, Chawade A (2021). Genome-wide association analysis and genomic prediction for adult-plant resistance to septoria tritici blotch and powdery mildew in winter wheat. Front Genet.

[34] Louriki S, Rehman S, El Hanafi S, Bouhouch Y, Al-Jaboobi M, Amri A, Douira A, Tadesse W (2021). Identification of resistance sources and genome-wide association mapping of septoria tritici blotch resistance in spring bread wheat germplasm of ICARDA. Front Plant Sci.

[35] Mahboubi M, Talebi R, Mehrabi R, Naji AM, Maccaferi M, Kema GHJ (2022). Genetic analysis of novel resistance sources and genome-wide association mapping identified novel QTLs for resistance to Zymoseptoria tritici, the causal agent of septoria tritici blotch in wheat. J Appl Genetics.

[36] Yang N, Ovenden B, Baxter B, McDonald MC, Solomon PS, Milgate A (2022). Multi-stage resistance to *Zymoseptoria tritici* revealed by GWAS in an Australian bread wheat diversity panel. Front Plant Sci.

[37] Odilbekov F, Armoniené R, Koc A, Svensson J, Chawade A (2019). GWAS-assisted genomic prediction to predict resistance to septoria tritici blotch in nordic winter wheat at seedling stage. Front Genet.

[38] Hafeez AM, Chartrain L, Feng C, Cambon F, Clarke M, Griffiths S, Hayta S, Jiang M, Keller B, Kirby R, Kolodziej MC, Powell OR, Smedley M, Steuernagel B, Xian W, Wingen LU, Cheng S, Saintenac C, Wulff BBH, Brown JKM. Septoria tritici blotch resistance gene Stb15 encodes a lectin receptor-like kinase. bioRxiv. 2023:09.11.557217. 10.1038/s41477-025-01920-2 PMC11928318 40087541

[39] Czembor PC, Radecka-Janusik M, Mańkowski D (2011). Virulence spectrum of *Mycosphaerella graminicola* isolates on wheat genotypes carrying known resistance genes to Septoria tritici blotch. J Phytopathol.

[40] Radecka-Janusik M, Czembor PC (2014). Genetic mapping of quantitative trait loci (QTL) for resistance to septoria tritici blotch in a winter wheat cultivar Liwilla. Euphytica.

[41] Kema GHJ, Annone JG, Sayoud R, VanSilfhout CH, VanGinkel M, deBree J (1996). Genetic variation for virulence and resistance in the wheat–*Mycosphaerella graminicola* pathosystem. 1. Interactions between pathogen isolates and host cultivars. Phytopathology.

[42] Tabib Ghaffary MT, Robert O, Laurent V, Lonnet P, Margale E, van der Lee TA, Visser RG, Kema GH (2011). Genetic analysis of resistance to Septoria tritici blotch in the French winter wheat cultivars Balance and Apache. Theor Appl Genet.

[43] Brinton J, Ramirez-Gonzalez RH, Simmonds J, Wingen L, Orford S, Griffiths S, Haberer G, Spannagl M, Walkowiak S, Pozniak C, Uauy C, 10 Wheat Genome Project (2020). A haplotype-led approach to increase the precision of wheat breeding. Commun Biol.

[44] Difabachew YF, Frisch M, Langstroff AL, Stahl A, Wittkop B, Snowdon RJ, Koch M, Kirchhoff M, Cselényi L, Wolf M, Förster J, Weber S, Okoye UJ, Zenke-Philippi C (2023). Genomic prediction with haplotype blocks in wheat. Front Plant Sci.

[45] Eriksen L, Borum F, Jahoor A (2003). Inheritance and localization of resistance to *Mycosphaerella graminicola* causing septoria tritici blotch and plant height in the wheat (*Triticum aestivum* L.) genome with DNA markers. Theor Appl Genet.

[46] Arraiano L, Balaam N, Fenwick P, Chapman C, Feuerhelm D, Howell P, Smith SJ, Widdowson JP, Brown JKM (2009). Contributions of disease resistance and escape to the control of Septoria tritici blotch of wheat. Plant Pathol.

[47] Flint-Garcia SA, Thornsberry JM, Buckler ES (2003). Structure of linkage disequilibrium in plants. Annu Rev Plant Biol.

[48] EnsemblPlants. https://plants.ensembl.org. Accessed 4 May 2023.

[49] Arraiano LS, Brown JK (2017). Sources of resistance and susceptibility to Septoria tritici blotch of wheat. Mol Plant Pathol.

[50] Yoshida T, Nishida H, Zhu J, Nitcher R, Distelfeld A, Akashi Y, Kato K, Dubcovsky J (2010). Vrn-D4 is a vernalization gene located on the centromeric region of chromosome 5D in hexaploid wheat. Theor Appl Genet.

[51] Simón MR, Worland AJ, Struik PC (2004). Influence of plant height and heading date on the expression of the resistance to septoria tritici blotch in near isogenic lines of wheat. Crop Sci.

[52] Simón MR, Perelló AE, Cordo CA, Larrán S, van der Putten PEL, Struik PC (2005). Association between septoria tritici blotch, plant height, and heading date in wheat. Agron J.

[53] Mądry W, Mańkowski DR, Kaczmarek Z, Krajewski P, Studnicki M (2010). Metody statystyczne oparte na modelach liniowych w zastosowaniach do doświadczalnictwa, genetyki i hodowli roślin. Monogr I Rozpr Nauk IHAR.

[54] Von Cruz M, Kilian A, Dierig DA (2013). Development of DArT marker platforms and genetic diversity assessment of the U.S. Collection of the new oilseed crop lesquerella and related species. PLoS ONE.

[55] Endelman JB (2011). Ridge regression and other kernels for genomic selection with R package rrBLUP. Plant Genome.

[56] Barrett JC, Fry B, Maller J, Daly MJ (2005). Haploview: analysis and visualization of LD and haplotype maps. Bioinformatics.

[57] Anand L, Rodriguez Lopez CM. ChromoMap: an R package for interactive visualization of multi-omics data and annotation of chromosomes. BMC Bioinformatics. 2022;23:33. 10.1186/s12859-021-04556-z PMC8753883 35016614

[58] Chambers JM. Graphical methods for data analysis, 1st ed. Chapman and Hall/CRC; 1983.

[59] Pritchard JK, Stephens M, Donnelly P (2000). Inference of population structure using multilocus genotype data. Genetics.

[60] Isidro-Sánchez J, Akdemir D, Montilla-Bascón G (2017). Genome-wide association analysis using R. Methods Mol Biol.

[61] Turner SD (2018). qqman: An R package for visualizing GWAS results using Q-Q and manhattan plots. J Open Source Softw.

[62] Lander E, Kruglyak L (1995). Genetic dissection of complex traits: guidelines for interpreting and reporting linkage results. Nat Genet.

[63] Konigorski S, Yilmaz YE, Janke J, Bergmann MM, Boeing H, Pischon T (2020). Powerful rare variant association testing in a copula-based joint analysis of multiple phenotypes. Gen Epidemiol.

