# Supplementary material for: Haplotype-based association mapping of genomic regions associated with Zymoseptoria tritici resistance using 217 diverse wheat genotypes
Source: BMC Plant Biol. 2024 Jul 18;24:682. doi: 10.1186/s12870-024-05400-1 (PMC11256644; doi:10.1186/s12870-024-05400-1)
Supplement: Supplementary file 3 — Supplementary Material 3. [file 12870_2024_5400_MOESM3_ESM.docx]

Haplotype-based association mapping of genomic regions associated with *Zymoseptoria tritici* resistance using 217 diverse wheat genotypes.

Magdalena Radecka-Janusik, Urszula Piechota, Dominika Piaskowska, Piotr Słowacki, Sławomir Bartosiak and Paweł Czembor

Table S3. Subpopulation Q-matrix according to the Structure software.

| **Subpopulation** | **Cultivar** | **Q1** | **Q2** | **Q3** | **Q4** |
| --- | --- | --- | --- | --- | --- |
| 1 | Kerubino | 1 | 0 | 0 | 0 |
| 1 | Zobel | 1 | 0 | 0 | 0 |
| 1 | Famulus | 0.999 | 0.001 | 0 | 0 |
| 1 | Arktis | 0.999 | 0 | 0 | 0 |
| 1 | Türkis | 0.999 | 0 | 0 | 0 |
| 1 | Rigi | 0.998 | 0.001 | 0 | 0 |
| 1 | Opal | 0.866 | 0.133 | 0 | 0 |
| 1 | Schamane | 0.821 | 0.174 | 0.005 | 0 |
| 1 | Sokrates | 0.816 | 0.178 | 0.005 | 0 |
| 1 | Wiwa | 0.799 | 0.09 | 0.002 | 0.11 |
| 1 | Kranich | 0.796 | 0 | 0.204 | 0 |
| 1 | Scaro | 0.784 | 0.102 | 0.003 | 0.111 |
| 1 | Pamier | 0.779 | 0 | 0.22 | 0 |
| 1 | Meteor | 0.776 | 0.001 | 0.223 | 0 |
| 1 | Praktik | 0.77 | 0.002 | 0.001 | 0.227 |
| 1 | Agil | 0.757 | 0.06 | 0.072 | 0.111 |
| 1 | Skagen | 0.742 | 0.001 | 0 | 0.257 |
| 1 | Butaro | 0.737 | 0.084 | 0.083 | 0.096 |
| 1 | Smaragd | 0.719 | 0.001 | 0.001 | 0.28 |
| 1 | Matrix | 0.717 | 0.041 | 0.072 | 0.17 |
| 1 | Kalahari | 0.709 | 0.042 | 0.069 | 0.18 |
| 1 | Garantus | 0.679 | 0.01 | 0.215 | 0.096 |
| 1 | Legenda | 0.626 | 0.036 | 0.337 | 0 |
| 1 | Dorota | 0.626 | 0.005 | 0.122 | 0.247 |
| 1 | Akteur | 0.598 | 0 | 0.401 | 0.001 |
| 1 | Naridana | 0.552 | 0.001 | 0.447 | 0 |
| 1 | Rywalka | 0.546 | 0 | 0.454 | 0 |
| 1 | Zeppelin | 0.537 | 0.098 | 0.137 | 0.228 |
| 1 | Bamberka | 0.529 | 0.001 | 0.001 | 0.47 |
| 1 | Nelson | 0.515 | 0.003 | 0.002 | 0.48 |
| 1 | Florian | 0.514 | 0.005 | 0.004 | 0.478 |
| 1 | Fregata | 0.501 | 0 | 0.498 | 0 |
| 1 | Zawisza | 0.5 | 0.029 | 0.394 | 0.077 |
| 1 | Patras | 0.371 | 0.002 | 0.319 | 0.307 |
| 1 | Fakir | 0.492 | 0.041 | 0.291 | 0.176 |
| 1 | Tengri | 0.458 | 0.269 | 0.148 | 0.124 |
| 2 | Cs Synthetic (6x) 7D | 0.001 | 0.999 | 0 | 0 |
| 2 | Chinese Spring | 0.001 | 0.999 | 0 | 0 |
| 2 | M6 synthetic (W-7984) | 0.051 | 0.949 | 0 | 0 |
| 2 | TE9111 | 0.091 | 0.908 | 0 | 0.001 |
| 2 | Veranopolis | 0.094 | 0.904 | 0.001 | 0.001 |
| 2 | Israel493 | 0.108 | 0.891 | 0 | 0 |
| 2 | M3 synthetic (W-7976) | 0.119 | 0.879 | 0.001 | 0.001 |
| 2 | Courtot | 0.105 | 0.877 | 0.001 | 0.017 |
| 2 | Taichung29 | 0.142 | 0.857 | 0 | 0 |
| 2 | Bulgaria88 | 0.153 | 0.846 | 0 | 0 |
| 2 | Estanzuela Federal | 0.16 | 0.84 | 0 | 0 |
| 2 | Mazurka | 0.22 | 0.728 | 0.05 | 0.003 |
| 2 | Salamouni | 0.154 | 0.712 | 0.092 | 0.042 |
| 2 | Ch Combin | 0.265 | 0.7 | 0.001 | 0.035 |
| 2 | Begra | 0.253 | 0.699 | 0.047 | 0 |
| 2 | MV Lucilla | 0.298 | 0.695 | 0.007 | 0 |
| 2 | Renan (RL 248) | 0.266 | 0.677 | 0.004 | 0.053 |
| 2 | Edelrun | 0.236 | 0.64 | 0.123 | 0 |
| 2 | Réciproc | 0.06 | 0.621 | 0.002 | 0.317 |
| 2 | Valdo | 0.001 | 0.621 | 0.001 | 0.377 |
| 2 | Tadinia | 0.235 | 0.58 | 0.185 | 0 |
| 2 | Oceano | 0.001 | 0.527 | 0.001 | 0.472 |
| 2 | Marcopolo | 0.001 | 0.517 | 0.102 | 0.38 |
| 2 | Jaceo | 0.001 | 0.503 | 0.141 | 0.355 |
| 2 | Alhambra | 0.064 | 0.489 | 0.278 | 0.168 |
| 2 | Baletka | 0.103 | 0.418 | 0.393 | 0.086 |
| 2 | Smuga | 0.274 | 0.408 | 0.317 | 0.001 |
| 2 | Ehogold 770/09 | 0.287 | 0.458 | 0.254 | 0.001 |
| 3 | Tulecka | 0 | 0 | 0.999 | 0 |
| 3 | Bockris | 0 | 0 | 0.999 | 0 |
| 3 | Sukces | 0.001 | 0 | 0.998 | 0 |
| 3 | Tonacja | 0.001 | 0.001 | 0.997 | 0.001 |
| 3 | Zyta | 0.003 | 0.001 | 0.995 | 0 |
| 3 | Nutka | 0.017 | 0.052 | 0.93 | 0 |
| 3 | Lahertis | 0.009 | 0 | 0.919 | 0.071 |
| 3 | Markiza | 0.009 | 0.082 | 0.909 | 0 |
| 3 | Bogatka | 0.05 | 0.057 | 0.893 | 0 |
| 3 | Figura | 0.008 | 0.018 | 0.878 | 0.096 |
| 3 | Batuta | 0.089 | 0.045 | 0.865 | 0 |
| 3 | Jantarka | 0.001 | 0.09 | 0.861 | 0.047 |
| 3 | Henrik | 0.001 | 0 | 0.854 | 0.145 |
| 3 | Astoria | 0.099 | 0 | 0.831 | 0.07 |
| 3 | Heros | 0.001 | 0.122 | 0.82 | 0.057 |
| 3 | Satyna | 0.043 | 0.005 | 0.807 | 0.144 |
| 3 | KWS Livius | 0.016 | 0 | 0.805 | 0.178 |
| 3 | Ostka Strzelecka | 0.181 | 0.016 | 0.804 | 0 |
| 3 | Kohelia | 0.19 | 0.008 | 0.801 | 0 |
| 3 | Platin | 0.01 | 0.001 | 0.793 | 0.197 |
| 3 | Arkadia | 0.001 | 0.001 | 0.786 | 0.212 |
| 3 | Glaucus | 0.077 | 0.006 | 0.775 | 0.143 |
| 3 | Estivus | 0.201 | 0.034 | 0.764 | 0.001 |
| 3 | Ludwig | 0.239 | 0.001 | 0.757 | 0.003 |
| 3 | Pengar | 0.146 | 0.096 | 0.75 | 0.008 |
| 3 | Eron | 0.201 | 0.051 | 0.748 | 0 |
| 3 | Fermi | 0.001 | 0.055 | 0.737 | 0.208 |
| 3 | Lavantus | 0.188 | 0.003 | 0.734 | 0.075 |
| 3 | Matheo | 0.001 | 0.134 | 0.733 | 0.133 |
| 3 | Capone | 0.007 | 0.157 | 0.727 | 0.108 |
| 3 | Tobak | 0.177 | 0.093 | 0.724 | 0.006 |
| 3 | Manager | 0.102 | 0.113 | 0.714 | 0.071 |
| 3 | Gordian | 0.287 | 0.003 | 0.709 | 0 |
| 3 | Desamo | 0.291 | 0 | 0.709 | 0 |
| 3 | Elixer | 0.114 | 0.114 | 0.702 | 0.07 |
| 3 | Mulan | 0.001 | 0 | 0.675 | 0.324 |
| 3 | Look | 0.014 | 0.001 | 0.673 | 0.312 |
| 3 | Pionier | 0.002 | 0.017 | 0.672 | 0.309 |
| 3 | Terroir | 0 | 0.334 | 0.658 | 0.007 |
| 3 | Solitar | 0.117 | 0.014 | 0.647 | 0.222 |
| 3 | Magnus | 0.17 | 0.144 | 0.632 | 0.054 |
| 3 | Bombus | 0.107 | 0.001 | 0.628 | 0.264 |
| 3 | Operetka | 0.004 | 0 | 0.622 | 0.374 |
| 3 | Dekan | 0.112 | 0.002 | 0.621 | 0.264 |
| 3 | Bystra | 0.001 | 0.09 | 0.609 | 0.3 |
| 3 | Sailor | 0.3 | 0.096 | 0.604 | 0.001 |
| 3 | Rumor | 0.198 | 0.082 | 0.595 | 0.125 |
| 3 | Kobiera | 0.247 | 0.159 | 0.593 | 0 |
| 3 | Askalon | 0.127 | 0 | 0.59 | 0.283 |
| 3 | Forkida | 0.212 | 0.115 | 0.587 | 0.086 |
| 3 | Bagou | 0.112 | 0 | 0.586 | 0.302 |
| 3 | Lear | 0.003 | 0.123 | 0.585 | 0.288 |
| 3 | Kredo | 0.064 | 0.108 | 0.585 | 0.244 |
| 3 | Joker | 0.194 | 0 | 0.583 | 0.222 |
| 3 | Memory | 0.375 | 0.046 | 0.568 | 0.011 |
| 3 | Belenus | 0.017 | 0.07 | 0.563 | 0.35 |
| 3 | Natula | 0.435 | 0.001 | 0.563 | 0.001 |
| 3 | Meister | 0.013 | 0 | 0.563 | 0.424 |
| 3 | Turnia | 0.189 | 0.16 | 0.561 | 0.09 |
| 3 | Primus | 0.08 | 0.032 | 0.547 | 0.341 |
| 3 | Julius | 0.36 | 0.048 | 0.537 | 0.055 |
| 3 | Ostroga | 0.056 | 0.104 | 0.533 | 0.307 |
| 3 | Discus | 0.266 | 0.073 | 0.529 | 0.132 |
| 3 | Artagnan | 0.001 | 0.108 | 0.513 | 0.378 |
| 3 | Belepi | 0.094 | 0.395 | 0.511 | 0 |
| 3 | Addict | 0.001 | 0.413 | 0.508 | 0.078 |
| 3 | Etana | 0.073 | 0.072 | 0.506 | 0.349 |
| 3 | Florett | 0.193 | 0.082 | 0.502 | 0.224 |
| 3 | KWS Dacanto | 0.294 | 0 | 0.394 | 0.311 |
| 3 | Forum | 0.289 | 0.001 | 0.433 | 0.277 |
| 3 | Speedway | 0.129 | 0.196 | 0.451 | 0.225 |
| 3 | Fidelius | 0.18 | 0.295 | 0.333 | 0.192 |
| 3 | Mandub | 0.073 | 0.333 | 0.447 | 0.147 |
| 3 | Wydma | 0.438 | 0.001 | 0.468 | 0.093 |
| 3 | Arina | 0.303 | 0.297 | 0.384 | 0.015 |
| 3 | Mikula | 0.246 | 0.254 | 0.499 | 0 |
| 3 | Mewa | 0.186 | 0.33 | 0.484 | 0 |
| 3 | Muza | 0.225 | 0.35 | 0.425 | 0 |
| 3 | Liwilla | 0.315 | 0.291 | 0.394 | 0 |
| 4 | Kampana | 0 | 0 | 0.001 | 0.999 |
| 4 | Muszelka | 0 | 0 | 0 | 0.999 |
| 4 | Alcazar | 0.001 | 0 | 0.004 | 0.995 |
| 4 | Kris | 0.001 | 0 | 0.058 | 0.941 |
| 4 | Rapsodia | 0.002 | 0.011 | 0.111 | 0.876 |
| 4 | Lithium | 0.003 | 0.005 | 0.119 | 0.874 |
| 4 | Waxy | 0.001 | 0 | 0.138 | 0.86 |
| 4 | Kepler | 0.142 | 0 | 0.014 | 0.844 |
| 4 | Olivin | 0.001 | 0.122 | 0.035 | 0.841 |
| 4 | Ionesco | 0.004 | 0.069 | 0.091 | 0.837 |
| 4 | Fructidor | 0.001 | 0.164 | 0.001 | 0.835 |
| 4 | Winnetou | 0.063 | 0.039 | 0.124 | 0.773 |
| 4 | Chilton | 0.118 | 0.055 | 0.054 | 0.773 |
| 4 | Samurai | 0.03 | 0.003 | 0.202 | 0.765 |
| 4 | Linus | 0.017 | 0 | 0.22 | 0.762 |
| 4 | Intro | 0.26 | 0.002 | 0 | 0.738 |
| 4 | RGT Frenezio | 0.003 | 0.278 | 0.001 | 0.718 |
| 4 | Celebration | 0.137 | 0.001 | 0.15 | 0.712 |
| 4 | Atomic | 0.034 | 0.012 | 0.244 | 0.711 |
| 4 | Barok | 0.055 | 0.228 | 0.007 | 0.71 |
| 4 | Sophytra | 0.235 | 0.065 | 0.004 | 0.696 |
| 4 | RGT Ampiezzo | 0.001 | 0.308 | 0 | 0.691 |
| 4 | Alchemy | 0.106 | 0.076 | 0.135 | 0.684 |
| 4 | Tuareg | 0.066 | 0.001 | 0.263 | 0.671 |
| 4 | Oxal | 0.283 | 0 | 0.052 | 0.665 |
| 4 | Mentor | 0.335 | 0.001 | 0.004 | 0.661 |
| 4 | Boomer | 0.092 | 0 | 0.256 | 0.651 |
| 4 | Gabrio | 0 | 0.251 | 0.116 | 0.633 |
| 4 | Xantippe | 0.081 | 0.11 | 0.182 | 0.627 |
| 4 | RGT Kilimanjaro | 0.351 | 0.005 | 0.02 | 0.624 |
| 4 | Avalon (W 2564) | 0.079 | 0.188 | 0.111 | 0.622 |
| 4 | Pueblo | 0.001 | 0.381 | 0 | 0.618 |
| 4 | Mandragor | 0.141 | 0.25 | 0.001 | 0.609 |
| 4 | Jenga | 0.026 | 0.001 | 0.386 | 0.587 |
| 4 | Eperon | 0.004 | 0.156 | 0.267 | 0.574 |
| 4 | Diamento | 0.001 | 0.427 | 0 | 0.572 |
| 4 | Starway | 0.036 | 0.017 | 0.378 | 0.569 |
| 4 | Banderola | 0.034 | 0.071 | 0.339 | 0.556 |
| 4 | Arezzo | 0.101 | 0.346 | 0 | 0.553 |
| 4 | Solognac | 0.016 | 0.437 | 0.002 | 0.546 |
| 4 | Colonia | 0.37 | 0.027 | 0.058 | 0.545 |
| 4 | Tentation | 0.001 | 0.16 | 0.304 | 0.534 |
| 4 | Frument | 0.001 | 0.097 | 0.376 | 0.525 |
| 4 | Edgar | 0.37 | 0.023 | 0.082 | 0.525 |
| 4 | Diderot | 0.115 | 0.285 | 0.08 | 0.521 |
| 4 | KWS Ozon | 0.314 | 0.003 | 0.17 | 0.513 |
| 4 | Elipsa | 0.004 | 0.151 | 0.334 | 0.512 |
| 4 | Caroll | 0.002 | 0.075 | 0.411 | 0.511 |
| 4 | Kantao | 0.061 | 0.298 | 0.132 | 0.509 |
| 4 | Zappa | 0.001 | 0.026 | 0.464 | 0.508 |
| 4 | Tabasco | 0.001 | 0.036 | 0.456 | 0.507 |
| 4 | Apache | 0.033 | 0.287 | 0.176 | 0.504 |
| 4 | Balance | 0.127 | 0.208 | 0.161 | 0.504 |
| 4 | Nocibe | 0.001 | 0.138 | 0.36 | 0.501 |
| 4 | Calcio | 0.001 | 0.473 | 0.025 | 0.501 |
| 4 | KWS Erasmus | 0.003 | 0.004 | 0.495 | 0.498 |
| 4 | Evolution | 0.006 | 0.151 | 0.349 | 0.495 |
| 4 | Thalys | 0.034 | 0.335 | 0.139 | 0.492 |
| 4 | Zephyr | 0.032 | 0.053 | 0.428 | 0.487 |
| 4 | Syllon | 0.422 | 0.092 | 0.002 | 0.485 |
| 4 | Avenir | 0.199 | 0.034 | 0.291 | 0.476 |
| 4 | Granamax | 0.102 | 0.18 | 0.255 | 0.464 |
| 4 | Torrild | 0.333 | 0.04 | 0.166 | 0.461 |
| 4 | Riband | 0.047 | 0.081 | 0.435 | 0.437 |
| 4 | Amifor | 0.178 | 0.275 | 0.131 | 0.416 |
| 4 | Flame | 0.328 | 0.16 | 0.1 | 0.412 |
| 4 | Grapeli | 0.174 | 0.136 | 0.293 | 0.398 |
| 4 | Dacanto | 0.336 | 0.001 | 0.276 | 0.388 |
| 4 | Salutos | 0.375 | 0.002 | 0.238 | 0.385 |
| 4 | Descartes | 0.246 | 0.266 | 0.123 | 0.365 |
| 4 | Artist | 0.227 | 0.001 | 0.413 | 0.359 |
| 4 | KWS Magic | 0.308 | 0.001 | 0.341 | 0.351 |
| 4 | Armada | 0.189 | 0.091 | 0.372 | 0.348 |
| 4 | RGT Djoko | 0.001 | 0.45 | 0.219 | 0.329 |

Table S5. Haplotypes described in MTA represented in 217 wheat genotypes. HB and HT in bold indicate sequence variant that decrease disease symptoms (NEC and/or PYC) according to box plots (Figure 6).

| **HB_HT** | **Cultivars** |
| --- | --- |
| Chr1A_HB86_HT9 | Mazurka, Tadinia, Estanzuela Federal, Akteur, Wydma, Kobiera, KWS Ozon, Zawisza, Dorota, Garantus, Turnia, Kepler, Smaragd, Diderot, Eron |
| Chr1B_HB123_HT1 | Agil, Alchemy, Alhambra, Amifor, Arezzo, Atomic, Avalon (W 2564), Avenir, Barok, Bombus, Butaro, Calcio, Ch Combin, Chilton, Colonia, Dacanto, Dekan, Descartes, Diamento, Discus, Edgar, Etana, Evolution, Florett, Florian, Fructidor, Frument, Gabrio, Glaucus, Intro, Jaceo, Kalahari, Kerubino, Magnus, Manager, Mandub, Mandragor, Marcopolo, Matrix, Mentor, Nelson, Oceano, Opal, Primus, Pueblo, Réciproc, Renan (RL 248), RGT Ampiezzo, RGT Frenezio, RGT Kilimanjaro, Riband, Rumor, Salutos, Samurai, Scaro, Schamane, Sokrates, Solognac, Sophytra, Starway, Syllon, Tengri, Tuareg, Valdo, Winnetou, Wiwa, Zeppelin, Zobel, Veranopolis, Israel493, Cs Synthetic (6x) 7D, M6 synthetic (W-7984), Courtot, TE9111, Salamouni, Arina, M3 synthetic (W-7976), Solitar, Apache, Chinese Spring, Taichung29, Begra, Akteur, Smuga, Wydma, Belepi, Baletka, Bystra, Forum, Kobiera, KWS Ozon, Meteor, Nutka, Platin, Alcazar, Speedway, Zawisza, Bamberka, Dorota, Fregata, Kohelia, Lavantus, Mewa, Olivin, Praktik, Arkadia, Sukces, Zyta, Capone, Banderola, Elipsa, Garantus, Kranich, Legenda, Mikula, Operetka, Rapsodia, Arktis, Tonacja, Addict, Caroll, Batuta, Estivus, Henrik, Kredo, Linus, Mulan, Ostka_Strzelecka, Rywalka, Artist, Torrild, Celebration, Belenus, Fakir, Jantarka, Kris, Look, Muszelka, Ostroga, Sailor, Askalon, Tulecka, Armada, Bockris, Fidelius, Jenga, KWS_Dacanto, Ludwig, Muza, Oxal, Satyna, Astoria, Türkis, Artagnan, Bogatka, Figura, Kampana, KWS Livius, Markiza, Naridana, Patras, Skagen, Bagou, Turnia, Boomer, Forkida, Kepler, KWS Magic, Meister, Natula, Pengar, Smaragd, Desamo, Heros, Nocibe, Zappa, Diderot, Ionesco, Lahertis, Pamier, Tabasco, Fermi, Joker, Lear, Pionier, Tentation, Elixer, Julius, Lithium, Terroir, Gordian, Kantao, RGT Djoko, Thalys, Eperon, Granamax, Matheo, Tobak, Grapeli, KWS Erasmus, Memory, Waxy |
| Chr1D_HB68_HT4 | Florian, Mazurka, Nelson, Schamane, Sokrates, Cs Synthetic (6x) 7D, Chinese Spring, Baletka, KWS Ozon, Bamberka, Dorota, Mewa, Garantus, Mulan, Fakir, Jantarka, Fidelius, Edelrun, Julius, MV Lucilla |
| Chr2A_HB11_HT23 | Butaro, Discus, Ehogold 770/09, Glaucus, Magnus, Mandub, Matrix, Riband, Rumor, Scaro, Schamane, Sokrates, Syllon, Wiwa, Zephyr, Zeppelin, Bulgaria88, Tadinia, Courtot, Arina, Balance, Begra, Smuga, Baletka, Nutka, Dorota, Lavantus, Arkadia, Zyta, Tonacja, Henrik, Torrild, Jantarka, Sailor, Ludwig, Muza, Satyna, Naridana, Turnia, Forkida, Heros, Tentation, Elixer, MV Lucilla, Thalys |
| Chr2B_HB25_HT3 | Etana, Scaro, Wiwa, Veranopolis, Estanzuela Federal, M6 synthetic (W-7984), M3 synthetic (W-7976), Chinese Spring, Taichung29, Akteur, Zawisza, Dorota, Arkadia, Rywalka, Artist, Skagen, Smaragd, Joker, Pionier, Thalys, Matheo |
| Chr2B_HB41_HT1 | Amifor, Israel493, Cs Synthetic (6x) 7D, M6 synthetic (W7984), TE9111, M3 synthetic (W-7976), Solitar, Chinese Spring, Belepi, Elipsa, KWS Livius, Memory |
| Chr2D_HB31_HT3 | Ehogold_770/09, Veranopolis, Cs Synthetic (6x) 7D, Estanzuela Federal, M6 synthetic (W-7984), Liwilla, Chinese Spring, Zawisza, Dorota, Zyta, Smaragd |
| Chr3A_HB68_HT1 | Ehogold_770/09, Veranopolis, Cs Synthetic (6x) 7D, Estanzuela Federal, M6 synthetic (W-7984), Chinese Spring, Taichung29, Begra, Smuga, Baletka, Fidelius, Edelrun, MV Lucilla |
| Chr3A_HB93_HT4 | Courtot, Taichung29, Wydma, Forum, Dorota, Banderola, Garantus, Arktis, Torrild, Ludwig, Smaragd, Memory, Eron |
| **Chr3A_HB98_HT2** | Agil, Alchemy, Amifor, Atomic, Avalon (W 2564), Avenir, Barok, Bombus, Butaro, Calcio, Ch Combin, Colonia, Dacanto, Dekan, Descartes, Diamento, Discus, Edgar, Famulus, Florett, Florian, Fructidor, Frument, Gabrio, Glaucus, Intro, Jaceo, Kerubino, Magnus, Mandub, Mandragor, Marcopolo, Matrix, Nelson, Oceano, Opal, PRIMUS, Pueblo, Réciproc, Renan (RL 248), RGT Ampiezzo, RGT Kilimanjaro, Riband, Rigi, Salutos, Samurai, Scaro, Schamane, Sokrates, Solognac, Sophytra, Starway, Syllon, Valdo, Winnetou, Zeppelin, Zobel, Israel493, Cs Synthetic (6x) 7D, Flame, TE9111, Salamouni, Arina, M3 synthetic (W-7976), Liwilla, Solitar, Apache, Chinese Spring, Taichung29, Akteur, Wydma, Belepi, Bystra, Kobiera, KWS Ozon, Meteor, Platin, Speedway, Zawisza, Bamberka, Fregata, Kohelia, Lavantus, Mewa, Olivin, Praktik, Arkadia, Sukces, Zyta, Capone, Banderola, Elipsa, Garantus, Kranich, Legenda, Operetka, Rapsodia, Arktis, Tonacja, Addict, Caroll, Estivus, Henrik, Kredo, Linus, Mulan, Ostka_Strzelecka, Rywalka, Artist, Celebration, Belenus, Fakir, Jantarka, Look, Muszelka, Ostroga, Sailor, Tulecka, Armada, Bockris, KWS Dacanto, Ludwig, Muza, Oxal, Satyna, Astoria, Türkis, Bogatka, Figura, KWS Livius, Markiza, Naridana, Patras, Bagou, Turnia, Boomer, Forkida, KWS Magic, Meister, Natula, Pengar, Desamo, Heros, Nocibe, Zappa, Diderot, Ionesco, Lahertis, Pamier, Tabasco, Fermi, Joker, Lear, Tentation, Elixer, Julius, Lithium, Terroir, Gordian, Kantao, RGT Djoko, Eperon, Granamax, Matheo, Tobak, Grapeli, KWS_Erasmus, Memory, Waxy |
| Chr3A_HB100_HT2 | Alchemy, Alhambra, Amifor, Ch Combin, Dekan, Discus, Ehogold 770/09, Florian, Frument, Kerubino, Magnus, MATRIX, Mazurka, Nelson, Pueblo, Renan (RL 248), Salutos, Starway, Syllon, Tengri, Zephyr, Bulgaria88, Estanzuela Federal, Arina, Liwilla, Balance, Begra, Akteur, Smuga, Wydma, Baletka, Zawisza, Mewa, Elipsa, Legenda, Mikula, Operetka, Arktis, Mulan, Celebration, Ostroga, Fidelius, Artagnan, Meister, Natula, Pengar, Heros, Edelrun, Fermi, Kantao, MV Lucilla, Thalys, Eperon, Tobak, Memory |
| Chr3B_HB123_HT3 | Chilton, Frument, Gabrio, RGT Ampiezzo, Rumor, Winnetou, TE9111, Forum, Meteor, Rapsodia, Belenus, Naridana, Pamier, Fermi, JOKER, Lear, RGT Djoko, Memory |
| Chr3D_HB11_HT4 | Alchemy, Avenir, Barok, Descartes, Matrix, Mazurka, Primus, Pueblo, RGT Ampiezzo, Riband, Solognac, Sophytra, Winnetou, Arina, Apache, Taichung29, Smuga, Alcazar, Bamberka, Dorota, Banderola, Rapsodia, Kredo, Linus, Mulan, Torrild, Celebration, Kris, Sailor, Jenga, Oxal, Satyna, Kampana, Skagen, Kepler, KWS Magic, Smaragd, Nocibe, Zappa, Lahertis, Tabasco, Fermi, Lear, Tentation, Eperon, KWS Erasmus |
| Chr4A_HB10_HT3 | Chilton, Magnus, Mandub, Rigi, Rumor, Salutos, Tengri, Bulgaria88, Taichung29, Arktis, Jantarka |
| Chr4A_HB74_HT2 | Mandub, Cs Synthetic (6x) 7D, Chinese Spring, Belepi, Speedway, Capone, Addict, Pengar, Heros, Zappa, Tabasco, Fermi, Lear, Elixer, Terroir, Gordian, RGT Djoko, Matheo, Tobak, Eron |
| Chr4B_HB68_HT1 | Agil, Alchemy, Alhambra, Amifor, Arezzo, Atomic, Avalon (W 2564), Avenir, Barok, Bombus, Butaro, Calcio, Ch Combin, Chilton, Colonia, Dacanto, Dekan, Descartes, Diamento, Discus, Edgar, Ehogold 770/09, Evolution, Famulus, Florett, Florian, Fructidor, Frument, Gabrio, Intro, Jaceo, Kalahari, Kerubino, Magnus, Manager, Mandub, Mandragor, Matrix, Mazurka, Mentor, Nelson, Oceano, Opal, Primus, Pueblo, Réciproc, Renan (RL 248), RGT Ampiezzo, RGT Frenezio, Riband, Rigi, Rumor, Salutos, Samurai, Scaro, Schamane, Sokrates, Solognac, Sophytra, Syllon, Tengri, Valdo, Winnetou, Wiwa, Zephyr, Tadinia, Cs Synthetic (6x) 7D, Estanzuela Federal, Courtot, TE9111, Arina, M3 synthetic (W-7976), Liwilla, Solitar, Balance, Chinese Spring, Taichung29, Begra, Akteur, Smuga, Wydma, Belepi, Baletka, Bystra, Forum, Kobiera, KWS Ozon, Meteor, Nutka, Platin, Alcazar, Speedway, Zawisza, Bamberka, Dorota, Fregata, Kohelia, Lavantus, Mewa, Olivin, Praktik, Arkadia, Sukces, Zyta, Capone, Banderola, Elipsa, Garantus, Kranich, Legenda, Mikula, Operetka, Rapsodia, Arktis, Tonacja, Addict, Caroll, Batuta, Estivus, Henrik, Kredo, Linus, Mulan, Ostka_Strzelecka, Rywalka, Artist, Torrild, Celebration, Belenus, Fakir, Jantarka, Kris, Look, Muszelka, Ostroga, Sailor, Askalon, Tulecka, Armada, Bockris, Fidelius, Jenga, KWS Dacanto, Ludwig, Muza, Oxal, Satyna, Astoria, Türkis, Artagnan, Bogatka, Figura, Kampana, KWS Livius, Markiza, Naridana, Patras, Skagen, Bagou, Turnia, Boomer, Forkida, Kepler, KWS Magic, Meister, Natula, Pengar, Smaragd, Desamo, Nocibe, Zappa, Diderot, Ionesco, Pamier, Tabasco, Edelrun, Fermi, Joker, Lear, Pionier, Tentation, Elixer, Julius, Lithium, Terroir, Gordian, Kantao, MV_Lucilla, RGT Djoko, Thalys, Eperon, Granamax, Matheo, Tobak, Grapeli, KWS Erasmus, Memory, Waxy |
| Chr5A_HB4_HT2 | Bombus, Calcio, Chilton, Dekan, Discus, Etana, Gabrio, Manager, Marcopolo, Matrix, Primus, RGT Frenezio, Tadinia, Taichung29, Baletka, Sukces, Elipsa, Operetka, Batuta, Mulan, Artist, Jantarka, Sailor, Turnia, Meister, Joker, Pionier, Kantao, Matheo, KWS Erasmus |
| Chr5A_HB29_HT1 | Agil, Alchemy, Alhambra, Amifor, Arezzo, Atomic, Avalon (W 2564), Avenir, Barok, Bombus, Calcio, Ch Combin, Chilton, Colonia, Dacanto, Dekan, Descartes, Discus, Edgar, Etana, Evolution, Famulus, Florett, Fructidor, Frument, Gabrio, Glaucus, Kerubino, Magnus, Manager, Mandub, Mandragor, Marcopolo, Matrix, Mentor, Oceano, Primus, Pueblo, Renan (RL 248), RGT Ampiezzo, RGT Frenezio, RGT Kilimanjaro, Rigi, Rumor, Salutos, Samurai, Scaro, Solognac, Sophytra, Starway, Syllon, Tengri, Tuareg, Valdo, Winnetou, Wiwa, Zephyr, Zeppelin, Zobel, Israel493, Tadinia, Flame, Estanzuela Federal, M6 synthetic (W-7984), Courtot, Arina, Liwilla, Solitar, Apache, Balance, Taichung29, Begra, Smuga, Wydma, Belepi, Baletka, Bystra, Forum, Kobiera, KWS Ozon, Meteor, Platin, Alcazar, Speedway, Zawisza, Bamberka, Dorota, Kohelia, Lavantus, Mewa, Olivin, Arkadia, Zyta, Capone, Banderola, Elipsa, Garantus, Legenda, Mikula, Operetka, Rapsodia, Arktis, Tonacja, Addict, Caroll, Batuta, Estivus, Henrik, Kredo, Linus, Mulan, Ostka_Strzelecka, Rywalka, Artist, Celebration, Belenus, Fakir, Jantarka, Kris, Look, Muszelka, Ostroga, Sailor, Askalon, Armada, Bockris, Fidelius, Jenga, KWS Dacanto, Ludwig, Muza, Oxal, Astoria, Türkis, Artagnan, Bogatka, Figura, Kampana, KWS Livius, Markiza, Naridana, Patras, Bagou, Turnia, Boomer, Forkida, Kepler, KWS Magic, Meister, Natula, Smaragd, Desamo, Heros, Nocibe, Zappa, Diderot, Ionesco, Lahertis, Tabasco, Edelrun, Fermi, Joker, Pionier, Tentation, Elixer, Julius, Lithium, Terroir, Gordian, Kantao, RGT Djoko, Thalys, Eperon, Granamax, Matheo, Tobak, Grapeli, KWS Erasmus, Memory, Waxy, Eron |
| Chr5A_HB29_HT6 | Butaro, Diamento, Ehogold 770/09, Florian, Intro, Jaceo, Kalahari, Nelson, Opal, Réciproc, Schamane, Sokrates, Bulgaria88, Veranopolis, TE9111, Salamouni, M3 synthetic (W-7976), Akteur, Nutka, Praktik, Sukces, Kranich, Torrild, Tulecka, Skagen, Pamier, MV Lucilla |
| **Chr5B_HB47_HT1** | Agil, Alchemy, Alhambra, Amifor, Arezzo, Atomic, Avalon (W 2564), Avenir, Barok, Bombus, Butaro, Calcio, Ch Combin, Chilton, Colonia, Dacanto, Dekan, Descartes, Diamento, Discus, Edgar, Ehogold 770/09, Etana, Evolution, Famulus, Florett, Florian, Fructidor, Frument, Gabrio, Glaucus, Intro, Jaceo, Kalahari, Kerubino, Magnus, Manager, Mandub, Mandragor, Marcopolo, Matrix, Mazurka, Mentor, Nelson, Oceano, Opal, Primus, Pueblo, Réciproc, Renan (RL 248), RGT Ampiezzo, RGT Frenezio, RGT Kilimanjaro, Riband, Rigi, Rumor, Salutos, Samurai, Scaro, Schamane, Sokrates, Solognac, Sophytra, Starway, Syllon, Tengri, Tuareg, Valdo, Winnetou, Wiwa, Zephyr, Zeppelin, Zobel, Bulgaria88, Israel493, Tadinia, Flame, Courtot, TE9111, Salamouni, Arina, M3 synthetic (W-7976), Liwilla, Solitar, Apache, Balance, Begra, Akteur, Wydma, Belepi, Baletka, Bystra, Forum, KWS Ozon, Meteor, Nutka, Platin, Alcazar, Speedway, Zawisza, Bamberka, Dorota, Fregata, Kohelia, Lavantus, Mewa, Olivin, Praktik, Arkadia, Sukces, Capone, Banderola, Elipsa, Garantus, Kranich, Legenda, Mikula, Operetka, Rapsodia, Arktis, Tonacja, Addict, Caroll, Batuta, Estivus, Henrik, Kredo, Linus, Mulan, Ostka Strzelecka, Rywalka, Artist, Torrild, Celebration, Belenus, Fakir, Jantarka, Kris, Look, Muszelka, Ostroga, Sailor, Askalon, Tulecka, Armada, Bockris, Fidelius, Jenga, KWS Dacanto, Ludwig, Muza, Oxal, Satyna, Astoria, Türkis, Artagnan, Bogatka, Figura, Kampana, KWS_Livius, Markiza, Naridana, Patras, Skagen, Bagou, Turnia, Boomer, Forkida, Kepler, KWS Magic, Meister, Natula, Smaragd, Desamo, Heros, Nocibe, Zappa, Diderot, Ionesco, Lahertis, Pamier, Tabasco, Fermi, JOKER, Lear, Pionier, Tentation, Elixer, Julius, Lithium, Terroir, Gordian, Kantao, MV Lucilla, RGT Djoko, Thalys, Eperon, Granamax, Matheo, Tobak, Grapeli, KWS Erasmus, Memory, Waxy, Eron |
| Chr5B_HB69_HT3 | Kalahari, Opal, Zeppelin, Bulgaria88, Cs Synthetic (6x) 7D, Estanzuela Federal, Chinese Spring, Taichung29, Smuga, Fakir, Skagen, Smaragd |
| Chr5B_B71_HT18 | Alhambra, Ehogold 770/09, Etana, Frument, Matrix, Primus, Veranopolis, Tadinia, Estanzuela Federal, M6 synthetic (W-7984), Liwilla, Solitar, Taichung29, Begra, Smuga, Platin, Kohelia, Mewa, Linus, Ostka Strzelecka, Rywalka, Ostroga, Ludwig, Muza, Bogatka, KWS Livius, Pengar, Memory, Eron |
| Chr5B_HB118_HT2 | Alhambra, Amifor, Ch Combin, Descartes, Diamento, Ehogold 770/09, Famulus, Florian, Gabrio, Jaceo, Marcopolo, Mazurka, Nelson, Réciproc, Renan (RL 248), RGT Ampiezzo, RGT Frenezio, Rigi, Solognac, Syllon, Valdo, Zephyr, Tadinia, Estanzuela Federal, Courtot, Apache, Balance, Taichung29, Begra, Wydma, Zawisza, Mewa, Sukces, Zyta, Operetka, Ostka Strzelecka, Muza, Naridana, Turnia, Diderot, Julius, MV Lucilla, Granamax, Grapeli |
| Chr5D_HB10_HT1 | Alchemy, Alhambra, Amifor, Arezzo, Atomic, Barok, Bombus, Butaro, Ch Combin, Dacanto, Dekan, Discus, Ehogold 770/09, Evolution, Famulus, Florett, Florian, Frument, Glaucus, Intro, Kalahari, Kerubino, Magnus, Manager, Mandub, Marcopolo, Matrix, Mazurka, Mentor, Nelson, Oceano, Opal, Primus, Pueblo, Réciproc, Renan (RL 248), RGT Kilimanjaro, Riband, Rigi, Rumor, Salutos, Samurai, Schamane, Sokrates, Sophytra, Starway, Syllon, Tengri, Valdo, Winnetou, Zephyr, Zeppelin, Zobel, Bulgaria88, Tadinia, Cs Synthetic (6x) 7D, Flame, Courtot, Salamouni, Liwilla, Solitar, Apache, Balance, Chinese Spring, Taichung29, Akteur, Smuga, Wydma, Belepi, Baletka, Bystra, Forum, Kobiera, KWS Ozon, Meteor, Nutka, Platin, Alcazar, Zawisza, Bamberka, Dorota, Fregata, Kohelia, Lavantus, Mewa, Praktik, Arkadia, Sukces, Zyta, Capone, Banderola, Elipsa, Garantus, Kranich, Legenda, Mikula, Operetka, Arktis, Tonacja, Addict, Caroll, Batuta, Estivus, Henrik, Kredo, Linus, Mulan, Ostka Strzelecka, Rywalka, Artist, Torrild, Celebration, Belenus, Fakir, Jantarka, Kris, Look, Muszelka, Ostroga, Sailor, Askalon, Tulecka, Armada, Bockris, Jenga, KWS Dacanto, Ludwig, Muza, Oxal, Satyna, Astoria, Türkis, Artagnan, Bogatka, Figura, Kampana, KWS Livius, Markiza, Naridana, Patras, Skagen, Bagou, Turnia, Boomer, Forkida, Kepler, KWS Magic, Meister, Natula, Pengar, Smaragd, Desamo, Heros, Nocibe, Zappa, Ionesco, Lahertis, Pamier, Tabasco, Edelrun, Fermi, Joker, Lear, Pionier, Tentation, Elixer, Julius, Lithium, Gordian, Kantao, MV Lucilla, Granamax, Matheo, Tobak, KWS Erasmus, Memory, Waxy, Eron |
| Chr5D_HB10_HT3 | Colonia, Diamento, Edgar, Fructidor, Tuareg, Israel493, Estanzuela Federal, TE9111, M3 synthetic (W7976), Diderot, Eperon |
| Chr5D_HB51_HT5 | Agil, Alchemy, Alhambra, Amifor, Arezzo, Atomic, Avalon (W 2564), Avenir, Barok, Bombus, Butaro, Calcio, Ch Combin, Chilton, Colonia, Dacanto, Dekan, Descartes, Diamento, Discus, Edgar, Ehogold 770/09, Etana, Evolution, Famulus, Florett, Florian, Fructidor, Frument, Gabrio, Glaucus, Intro, Jaceo, Kalahari, Kerubino, Magnus, Manager, Mandragor, Marcopolo, Matrix, Mazurka, Mentor, Nelson, Oceano, Opal, PRIMUS, Pueblo, Réciproc, Renan (RL 248), RGT Ampiezzo, RGT Frenezio, RGT Kilimanjaro, Riband, Rigi, Rumor, Salutos, Samurai, Scaro, Schamane, Sokrates, Solognac, Sophytra, Starway, Syllon, Tengri, Tuareg, Valdo, Winnetou, Wiwa, Zephyr, Zeppelin, Zobel, Bulgaria88, Israel493, Flame, Estanzuela Federal, Courtot, TE9111, Arina, Liwilla, Solitar, Apache, Balance, Begra, Akteur, Smuga, Wydma, Baletka, Forum, Kobiera, KWS Ozon, Meteor, Nutka, Platin, Alcazar, Zawisza, Bamberka, Dorota, Fregata, Kohelia, Lavantus, Mewa, Olivin, Praktik, Arkadia, Sukces, Zyta, Banderola, Elipsa, Garantus, Kranich, Legenda, Mikula, Operetka, Rapsodia, Arktis, Tonacja, Caroll, Batuta, Estivus, Henrik, Linus, Mulan, Ostka Strzelecka, Rywalka, Artist, Torrild, Celebration, Belenus, Fakir, Jantarka, Kris, Look, Muszelka, Ostroga, Sailor, Askalon, Tulecka, Armada, Bockris, Fidelius, Jenga, KWS Dacanto, Ludwig, Muza, Oxal, Satyna, Astoria, Türkis, Artagnan, Bogatka, Figura, Kampana, KWS Livius, Markiza, Naridana, Patras, Skagen, Bagou, Turnia, Boomer, Forkida, Kepler, KWS Magic, Meister, Natula, Smaragd, Desamo, Nocibe, Diderot, Ionesco, Lahertis, Pamier, Edelrun, Joker, Pionier, Tentation, Julius, Lithium, Kantao, MV Lucilla, Thalys, Eperon, Granamax, KWS Erasmus, Memory, Waxy |
| **Chr6B_HB31_HT2** | Alhambra, Barok, Ch Combin, Dekan, Discus, Florian, Frument, Mandub, Nelson, Tuareg, Bulgaria88, Veranopolis, Israel493, Cs Synthetic (6x) 7D, Estanzuela Federal, M6 synthetic (W-7984), Courtot, TE9111, Arina, Liwilla, Solitar, Chinese Spring, Belepi, Bystra, Speedway, Capone, Mikula, Addict, Kredo, Celebration, Jenga, Pengar, Heros, Lear, Elixer, Lithium, Gordian, Kantao, RGT Djoko, Grapeli, Eron |
| Chr7A_HB107_HT3 | Alhambra, Florett, Jaceo, Mandub, Solognac, Israel493, Cs Synthetic (6x) 7D, Estanzuela Federal, TE9111, M3 synthetic (W-7976), Begra, Nutka, Zyta, Capone, Mikula, Tonacja, Addict, Kredo, Belenus, Tulecka, Muza, Oxal, Bogatka, Markiza, Naridana, Kepler, Pengar, Desamo, Heros, Diderot, Fermi, Terroir, RGT Djoko, Tobak, Grapeli, Eron |
| **Chr7B_HB36_HT1** | Agil, Alchemy, Alhambra, Amifor, Arezzo, Atomic, Avalon (W 2564), Avenir, Barok, Bombus, Butaro, Calcio, Ch Combin, Chilton, Colonia, Dacanto, Dekan, Descartes, Diamento, Discus, Edgar, Ehogold 770/09, Evolution, Famulus, Florett, Frument, Gabrio, Glaucus, Intro, Jaceo, Kalahari, Kerubino, Magnus, Manager, Mandub, Marcopolo, Matrix, Mazurka, Mentor, Oceano, Opal, Primus, Réciproc, Renan (RL 248), RGT Ampiezzo, RGT Frenezio, RGT Kilimanjaro, Riband, Rigi, Rumor, Salutos, Samurai, Scaro, Solognac, Sophytra, Starway, Syllon, Tengri, Tuareg, Valdo, Winnetou, Wiwa, Zephyr, Zeppelin, Zobel, Bulgaria88, Tadinia, Flame, M6 synthetic (W-7984), Courtot, TE9111, Arina, Liwilla, Solitar, Apache, Balance, Taichung29, Begra, Akteur, Wydma, Belepi, Baletka, Bystra, Forum, Kobiera, KWS Ozon, Meteor, Nutka, Platin, Alcazar, Speedway, Zawisza, Fregata, Kohelia, Lavantus, Mewa, Praktik, Arkadia, Sukces, Zyta, Capone, Banderola, Elipsa, Kranich, Legenda, Mikula, Operetka, Rapsodia, Arktis, Tonacja, Addict, Caroll, Batuta, Estivus, Henrik, Kredo, Linus, Mulan, Ostka_Strzelecka, Rywalka, Artist, Torrild, Celebration, Belenus, Fakir, Jantarka, Kris, Look, Muszelka, Ostroga, Sailor, Askalon, Tulecka, Armada, Bockris, Fidelius, Jenga, KWS Dacanto, Ludwig, Muza, Oxal, Satyna, Astoria, Türkis, Artagnan, Bogatka, Figura, Kampana, KWS Livius, Markiza, Naridana, Patras, Bagou, Turnia, Boomer, Forkida, Kepler, KWS Magic, Meister, Natula, Pengar, Desamo, Heros, Nocibe, Zappa, Diderot, Ionesco, Lahertis, Pamier, Tabasco, Edelrun, Fermi, Joker, Lear, Pionier, Tentation, Elixer, Julius, Lithium, Terroir, Gordian, Kantao, MV Lucilla, RGT Djoko, Thalys, Eperon, Granamax, Matheo, Tobak, Grapeli, KWS Erasmus, Memory, Waxy, Eron |

Table S7. The set of 22 wheat cultivars/lines with known or postulated resistance genes and susceptible checks used in GWAS analysis for Septoria tritici blotch resistance.

| **No.** | **Cultivar name** | **STB resistance genes** | **Source of information on STB resistance*** |
| --- | --- | --- | --- |
| 1 | Apache | *Stb11*(1BS), *Stb4*(7DS), *Stb5*(7DS), QTL–7DL | Tabib Ghaffary et al. (2011) |
| 2 | Arina | *Stb15*(6AS), *Stb6*(3AS) | Arraiano et al. (2007), Chartrain et al. (2005a) |
| 3 | Balance | *Stb18*(6DS) | Tabib Ghaffary et al.(2011) |
| 4 | Bulgaria88 | *Stb1*(5BL), *Stb6*(3AS) | Adhikari et al. (2004a), Chartrain et al.(2005a) |
| 5 | Chinese Spring | *Stb6*(3AS) | Brading et al. (2002), Chartrain et al. (2005a) |
| 6 | Courtot | *Stb9*(2BL) | Chartrain et al. (2009) |
| 7 | Cs Synthetic (6x)7D | *Stb5*(7DS), *Stb6*(3AS) | Arraiano et al. (2001) |
| 8 | Estanzuela Federal | *Stb7*(4AL) | McCartney et al. (2003) |
| 9 | Flame | *Stb6*(3AS) | Brading et al. (2002), Chartrain et al. (2005a) |
| 10 | Florett | *Stb6*(3AS), *Stb15*(6AS), QTL – 3B, 6D | Risser et al. (2011) |
| 11 | Israel493 | *Stb3*(7AS), *Stb6*(3AS) | Goodwin et al. (2015), Chartrain et al. (2005a) |
| 12 | Liwilla | *Stb6*(3AS), *Stb2*/*Stb11*(1BS), QTL – 6A, 7A, 2B, 3B, 5D | Radecka–Janusik and Czembor (2014) |
| 13 | M3 synthetic (W-7976) | *Stb16q*(3DL), *Stb17*(5AL) | Tabib Ghaffary et al. (2012) |
| 14 | M6 synthetic (W-7984) | *Stb8*(7BL) | Adhikari et al. (2003) |
| 15 | Mazurka | *Stb7*/*Stb12*(4AL) | Kelm et al. (2012) |
| 16 | Riband | QTL – 6B | Chartrain et al. (2004) |
| 17 | Salamouni | *Stb13*(7BL), *Stb14*(3BS) | KOMUGI (2021) |
| 18 | Solitar | *Stb6*(3AS), *Stb11*(1BS), QTL – 5A, 1B, 6B, 3D, 6D, 7D | Kelm et al. (2012) |
| 19 | Tadinia | *Stb4*(7DS), *Stb6*(3AS) | Adhikari et al. (2004b), Chartrain et al. (2005a) |
| 20 | TE9111 | *Stb11*(1BS), *Stb6*(3AS), *Stb7*(4AL) | Chartrain et al. (2005b) |
| 21 | Tuareg | *Stb6*(3AS), QTL – 4B, 6B | Risser et al. (2011) |
| 22 | Veranopolis | *Stb2*(1BS), *Stb6*(3AS) | Liu et al. (2013), Chartrain et al. (2005a) |
| 23 | Taichung29 | susceptible check | Tabib Ghaffary et al. (2012) |
| 24 | Begra | susceptible check | Czembor et al. (2011), Radecka–Janusik and Czembor (2014) |

* Table reference list:

Tabib Ghaffary MT, Robert O, Laurent V, Lonnet P, Margale ́ E, van der Lee TA, Visser RG, Kema GH. Genetic analysis of resistance to Septoria tritici blotch in the French winter wheat cultivars Balance and Apache. Theor Appl Genet 2011;123:741–754.

Arraiano LS, Chartrain L, Bossolini E, Slatter HN, Keller B, Brown JKM. A gene in European wheat cultivars for resistance to an African isolate of Mycosphaerella graminicola. Plant Pathol. 2007;56:73–78.

Chartrain L, Berry ST, Brown JKM. Resistance of wheat line Kavkaz- K4500 L.6.A.4 to Septoria tritici blotch controlled by isolate-specific resistance genes. Phytopathology 2005a;95:664–671.

Adhikari TB, Yang X, Cavaletto JR, Hu X, Buechley G, Ohm HW, Shaner G, Goodwin SB. Molecular mapping of Stb1, a potentially durable gene for resistance to Septoria tritici blotch in wheat. Theor Appl Genet. 2004a;109:944–953.

Brading PA, Verstappen ECP, Kema GHJ, Brown JKM. A gene-for-gene relationship between wheat and Mycosphaerella graminicola, the Septoria tritici blotch pathogen. Phytopathology. 2002;92:439–445.

Chartrain L, Sourdille P, Bernard M, Brown JKM. Identification and location of Stb9, a gene for resistance to Septoria tritici blotch in wheat cultivars Courtot and Tonic. Plant Pathol. 2009;58: 547–555.

Arraiano LS, Worland AJ, Ellerbrook C, Brown JKM. Chromosomal location of a gene for resistance to Septoria tritici blotch (Mycosphaerella graminicola) in the hexaploid wheat ‘Synthetic 6x’. Theor Appl Genet. 2001;103:758–764.

McCartney CA, Brûlé-Babel AL, Lamari L, Somers DJ. Chromosomal location of a race specific resistance gene to Mycosphaerella graminicola in the spring wheat ST6. Theor Appl Genet. 2003;107:1181–1186.

Risser P, Ebmeyer E, Korzun V, Hartl L, Miedaner T. Quantitative trait loci for adult-plant resistance to Mycosphaerella graminicola in two winter wheat populations. Phytopathology. 2011;101:1209–1216.

Goodwin SB, Cavaletto JR, Hale IL, Thompson I, Xu SX, Adhikari T B, Dubcovsky J. A New Map Location of Gene Stb3 for Resistance to Septoria Tritici Blotch in Wheat. Crop Sci. 2015;55(1):35-43.

Radecka-Janusik M, Czembor PC. Genetic mapping of quantitative trait loci (QTL) for resistance to septoria tritici blotch in a winter wheat cultivar Liwilla. Euphytica. 2014;200:109–125.

Tabib Ghaffary SM, Faris JD, Friesen TL, Visser RGF, van der Lee TAJ, Robert O, Kema GHJ. New broad-spectrum resistance to Septoria tritici blotch derived from synthetic hexaploid wheat. Theor Appl Genet. 2012;124: 125–142.

Adhikari TB, Anderson JM, Goodwin SB. Identification and molecular mapping of a gene in wheat conferring resistance to Mycosphaerella graminicola. Phytopathology 2003;93:1158-1164.

Kelm C, Tabib Ghaffary SM, Bruelheide H, Order MS, Miersch S, Weber WE, Kema GHJ, Saal B. The genetic architecture of seedling resistance to Septoria tritici blotch in the winter wheat doubled-haploid population Solitär x Mazurka. Mol Breed. 2012;29:813–830.

Chartrain L, Brading PA, Widdowson JP, Brown JKM. Partial resistance to Septoria tritici blotch (Mycosphaerella graminicola) in the wheat cultivars Arina and Riband. Phytopathology. 2004;94:497–504.

KOMUGI, Wheat Genetic Resources Database, https://shigen.nig.ac.jp/wheat/komugi/genes/symbolClassList.jsp. Accessed December 2021.

Adhikari TB, Cavaletto JR, Dubcovsky J, Gieco JO, Schlatter AR, Goodwin SB. Molecular mapping of the Stb4 gene for resistance to Septoria tritici blotch in wheat. Phytopathology. 2004b;94:1198–1206.

Chartrain L, Joaquim P, Berry ST, Arraiano LS, Azanza F, Brown JKM. Genetics of resistance to Septoria tritici blotch in the Portuguese wheat breeding line TE9111. Theor Appl Genet. 2005b;110:1138–1144.

Liu Y, Zhang L, Thompson IA, Goodwin SB, Ohm HW. Molecular mapping re-locates the Stb2 gene for resistance to Septoria tritici blotch derived from cultivar Veranopolis on wheat chromosome 1BS. Euphytica. 2013;190:145–156.

Czembor PC, Radecka-Janusik M, Mańkowski D. Virulence spectrum of Mycosphaerella graminicola isolates on wheat genotypes carrying known resistance genes to Septoria tritici blotch. J Phytopathol. 2011;159:146–154.

Table S8. The set of 217 wheat cultivars/lines used in GWAS analysis for Septoria tritici blotch resistance.

| **No.** | **Cultivar name** | **Country** | **Cultivar breeder/maintainer** | **Year of registration** | **Source of information on STB resistance**** |
| --- | --- | --- | --- | --- | --- |
| 1 | Arkteur | Germany | Deutsche Saatveredelung AG | 2007 | COBORU, 2014 (Poland) |
| 2 | Alcazar | Poland | DANKO Hodowla Roślin sp. z o. o. | 2006 | COBORU, 2014 (Poland) |
| 3 | Arkadia | Poland | DANKO Hodowla Roślin sp. z o. o. | 2011 | COBORU, 2014 (Poland) |
| 4 | Arktis | Germany | Deutsche Saatveredelung AG | 2013 | COBORU, 2014 (Poland) |
| 5 | Artist | Germany | Deutsche Saatveredelung AG | 2013 | COBORU, 2014 (Poland) |
| 6 | Askalon | Germany | Nordsaat Saatzucht GmbH | 2009 | COBORU, 2014 (Poland) |
| 7 | Astoria | Poland | Poznańska Hodowla Roślin sp. z o.o. | 2012 | COBORU, 2014 (Poland) |
| 8 | Bagou | France | Saaten Union Recherche | 2009 | COBORU, 2014 (Poland) |
| 9 | Baletka | France | RAGT 2n | 2009 | COBORU, 2014 (Poland) |
| 10 | Bamberka | Poland | Hodowla Roślin Strzelce sp. z o. o. Grupa IHAR | 2009 | COBORU, 2014 (Poland) |
| 11 | Banderola | Poland | DANKO Hodowla Roślin sp. z o. o. | 2010 | COBORU, 2014 (Poland) |
| 12 | Batuta | Poland | DANKO Hodowla Roślin sp. z o. o. | 2006 | COBORU, 2014 (Poland) |
| 13 | Belenus | France | RAGT 2n | 2009 | COBORU, 2014 (Poland) |
| 14 | Bockris | Germany | Strube Research GmbH & Co. KG | 2010 | COBORU, 2014 (Poland) |
| 15 | Bogatka | Poland | DANKO Hodowla Roślin sp. z o. o. | 2004 | COBORU, 2014 (Poland) |
| 16 | Boomer | Germany | Dieckmann GmbH & Co KG | 2006 | COBORU, 2014 (Poland) |
| 17 | Bystra | France | RAGT 2n | 2009 | COBORU, 2014 (Poland) |
| 18 | Dorota | France | RAGT 2n | 2004 | COBORU, 2014 (Poland) |
| 19 | Elipsa | Belgium | Limagrain GmbH | 2011 | COBORU, 2014 (Poland) |
| 20 | Estivus | Germany | Strube Research GmbH &C0.KG | 2012 | COBORU, 2014 (Poland) |
| 21 | Fakir | Germany | Syngenta Seeds GmbH | 2013 | COBORU, 2014 (Poland) |
| 22 | Fidelius | Austria | Saatzucht Donau Ges.m.b.H. & Co.KG | 2010 | COBORU, 2014 (Poland) |
| 23 | Figura | Poland | DANKO Hodowla Roślin sp. z o. o. | 2007 | COBORU, 2014 (Poland) |
| 24 | Forkida | Poland | DANKO Hodowla Roślin sp. z o. o. | 2010 | COBORU, 2014 (Poland) |
| 25 | Forum | Germany | Nordsaat Saatzucht GmbH | 2012 | COBORU, 2014 (Poland) |
| 26 | Fregata | Poland | Hodowla Roślin Strzelce sp. z o. o. Grupa IHAR | 2004 | COBORU, 2014 (Poland) |
| 27 | Garantus | France | RAGT 2n | 2007 | COBORU, 2014 (Poland) |
| 28 | Henrik | Germany | Limagrain GmbH | 2010 | COBORU, 2014 (Poland) |
| 29 | Jantarka | Poland | DANKO Hodowla Roślin sp. z o. o. | 2010 | COBORU, 2014 (Poland) |
| 30 | Jenga | Germany | Ackermann Saatzucht GmbH & Co. KG | 2008 | COBORU, 2014 (Poland) |
| 31 | Kampana | Poland | DANKO Hodowla Roślin sp. z o. o. | 2009 | COBORU, 2014 (Poland) |
| 32 | Kepler | Germany | Limagrain GmbH | 2010 | COBORU, 2014 (Poland) |
| 33 | Kobiera | Poland | Małopolska Hodowla Roślin - HBP sp. z o. o. | 2003 | COBORU, 2014 (Poland) |
| 34 | Kohelia | Poland | Małopolska Hodowla Roślin - HBP sp. z o. o. | 2008 | COBORU, 2014 (Poland) |
| 35 | Kranich | Germany | Lantmännen SW Seed GmbH | 2009 | COBORU, 2014 (Poland) |
| 36 | Kredo | Germany | Nordsaat Saatzucht GmbH | 2010 | COBORU, 2014 (Poland) |
| 37 | Kris | France | RAGT 2n | 2000 | COBORU, 2014 (Poland) |
| 38 | KWS Dacanto | Germany | KWS Lochow GmbH | 2011 | COBORU, 2014 (Poland) |
| 39 | KWS Livius | Germany | KWS Lochow GmbH | 2013 | COBORU, 2014 (Poland) |
| 40 | KWS Magic | Germany | KWS Lochow GmbH | 2012 | COBORU, 2014 (Poland) |
| 41 | KWS Ozon | Germany | KWS Lochow GmbH | 2010 | COBORU, 2014 (Poland) |
| 42 | Lavantus | Germany | Strube Research GmbH & Co. KG | 2013 | COBORU, 2014 (Poland) |
| 43 | Legenda | Poland | Poznańska Hodowla Roślin sp. z o.o. | 2005 | COBORU, 2014 (Poland) |
| 44 | Linus | France | RAGT 2n | 2011 | COBORU, 2014 (Poland) |
| 45 | Look | Germany | Dieckmann GmbH & Co KG | 2009 | COBORU, 2014 (Poland) |
| 46 | Ludwig | Poland | DANKO Hodowla Roślin sp. z o. o. | 2006 | COBORU, 2014 (Poland) |
| 47 | Markiza | Poland | Hodowla Roślin Strzelce sp. z o. o. Grupa IHAR | 2007 | COBORU, 2014 (Poland) |
| 48 | Meister | France | RAGT 2n | 2011 | COBORU, 2014 (Poland) |
| 49 | Meteor | Germany | Syngenta Seeds GmbH | 2007 | COBORU, 2014 (Poland) |
| 50 | Mewa | Poland | DANKO Hodowla Roślin sp. z o. o. | 1998 | COBORU, 2014 (Poland) |
| 51 | Mikula | Poland | Małopolska Hodowla Roślin - HBP sp. z o. o. | 1999 | COBORU, 2014 (Poland) |
| 52 | Mulan | Germany | Nordsaat Saatzucht GmbH | 2008 | COBORU, 2014 (Poland) |
| 53 | Muszelka | Poland | DANKO Hodowla Roślin sp. z o. o. | 2008 | COBORU, 2014 (Poland) |
| 54 | Muza | Poland | Małopolska Hodowla Roślin - HBP sp. z o. o. | 2004 | COBORU, 2014 (Poland) |
| 55 | Naridana | Poland | Poznańska Hodowla Roślin sp. z o.o. | 2006 | COBORU, 2014 (Poland) |
| 56 | Natula | Poland | Małopolska Hodowla Roślin - HBP sp. z o. o. | 2009 | COBORU, 2014 (Poland) |
| 57 | Nutka | Poland | Hodowla Roślin Strzelce sp. z o. o. Grupa IHAR | 2001 | COBORU, 2014 (Poland) |
| 58 | Olivin | France | RAGT 2n | 2004 | COBORU, 2014 (Poland) |
| 59 | Operetka | France | Nickerson International Research SNC | 2010 | COBORU, 2014 (Poland) |
| 60 | Ostka Strzelecka | Poland | Hodowla Roślin Strzelce sp. z o. o. Grupa IHAR | 2006 | COBORU, 2014 (Poland) |
| 61 | Ostroga | Poland | DANKO Hodowla Roślin sp. z o. o. | 2008 | COBORU, 2014 (Poland) |
| 62 | Oxal | France | RAGT 2n | 2011 | COBORU, 2014 (Poland) |
| 63 | Patras | France | Secobra Recherches | 2012 | COBORU, 2014 (Poland) |
| 64 | Pengar | Germany | W. von Borries-Eckendorf GmbH & Co. KG | 2013 | COBORU, 2014 (Poland) |
| 65 | Platin | Germany | Strube Research GmbH & Co. KG | 2012 | COBORU, 2014 (Poland) |
| 66 | Praktik | France | RAGT 2n | 2012 | COBORU, 2014 (Poland) |
| 67 | Rapsodia | France | RAGT 2n | 2003 | COBORU, 2014 (Poland) |
| 68 | Rywalka | Poland | Hodowla Roślin Strzelce sp. z o. o. Grupa IHAR | 2003 | COBORU, 2014 (Poland) |
| 69 | Sailor | France | Secobra Recherches | 2011 | COBORU, 2014 (Poland) |
| 70 | Satyna | Poland | Małopolska Hodowla Roślin - HBP sp. z o. o. | 2004 | COBORU, 2014 (Poland) |
| 71 | Skagen | Germany | W. von Borries-Eckendorf GmbH & Co. KG | 2009 | COBORU, 2014 (Poland) |
| 72 | Smaragd | Germany | SW Seed GmbH | 2009 | COBORU, 2014 (Poland) |
| 73 | Smuga | Poland | DANKO Hodowla Roślin sp. z o. o. | 2004 | COBORU, 2014 (Poland) |
| 74 | Speedway | Germany | Nordsaat Saatzucht GmbH | 2012 | COBORU, 2014 (Poland) |
| 75 | Sukces | Poland | Hodowla Roślin Strzelce sp. z o. o. Grupa IHAR | 2001 | COBORU, 2014 (Poland) |
| 76 | Tonacja | Poland | Hodowla Roślin Strzelce sp. z o. o. Grupa IHAR | 2001 | COBORU, 2014 (Poland) |
| 77 | Torrild | Germany | W. von Borries-Eckendorf GmbH & Co. KG | 2010 | COBORU, 2014 (Poland) |
| 78 | Tulecka | Poland | Poznańska Hodowla Roślin sp. z o.o. | 2012 | COBORU, 2014 (Poland) |
| 79 | Türkis | Germany | Lantmännen SW Seed GmbH | 2006 | COBORU, 2014 (Poland) |
| 80 | Turnia | Poland | Małopolska Hodowla Roślin - HBP sp. z o. o. | 2001 | COBORU, 2014 (Poland) |
| 81 | Wydma | Poland | Hodowla Roślin Smolice sp. z o. o. Grupa IHAR | 2005 | COBORU, 2014 (Poland) |
| 82 | Zawisza | Poland | Hodowla Roślin Smolice sp. z o. o. Grupa IHAR | 2004 | COBORU, 2014 (Poland) |
| 83 | Zyta | Poland | Hodowla Roślin Strzelce sp. z o. o. Grupa IHAR | 1999 | COBORU, 2014 (Poland) |
| 84 | Addict | France | Lemaire Deffontaines | 2011 | GEVES, 2013 (France) |
| 85 | Agil | Lithuania | Syngenta Seeds GmbH | 2004 | Bundessortenamt, 2013 (Germany) |
| 86 | Alchemy | Great Britain | Limagrain UK | 2005 | HGCA 2014-2015 (Great Britain) |
| 87 | Alhambra | France | Limagrain GmbH | 2013 | GEVES, 2013 (France) |
| 88 | Amifor | France | Unisigma | 2013 | GEVES, 2013 (France) |
| 89 | Arezzo | France | RAGT 2n | 2007 | GEVES, 2013 (France) |
| 90 | Armada | France | Limagrain Europe | 2011 | GEVES, 2013 (France) |
| 91 | Artagnan | France | Limagrain Europe | 2012 | GEVES, 2013 (France) |
| 92 | Atomic | Germany | Limagrain GmbH | 2012 | Bundessortenamt, 2013 (Germany) |
| 93 | Avalon (W 2564) | Great Britain | NA* | 1980 | John Innes Centre, 2013 (UK) |
| 94 | Avenir | Germany | Saatzucht Josef Breun | 2013 | Bundessortenamt, 2013 (Germany) |
| 95 | Barok | France | Agri Obtentions SA | 2009 | Bundessortenamt, 2013 (Germany) |
| 96 | Belepi | France | Lemaire Deffontaines | 2011 | GEVES, 2013 (France) |
| 97 | Bombus | Germany | Secobra Recherches | 2012 | Bundessortenamt, 2013 (Germany) |
| 98 | Butaro | Germany | Landbauschule Dottenfelderhof e.V./Spieß | 2009 | Bundessortenamt, 2013 (Germany) |
| 99 | Calcio | France | RAGT 2n | 2011 | GEVES, 2013 (France) |
| 100 | Capone | Germany | Limagrain GmbH | 2012 | Bundessortenamt, 2013 (Germany) |
| 101 | Caroll | Netherlands | B.V. Landbouwbureau Wiersum | 2011 | ÚKZÚZ, 2013 (Czech Republik) |
| 102 | Celebration | Germany | NA | NA | Deutsche Saatveredelung AG, 2013 (Germany) |
| 103 | Ch Combin | Switzerland | Delley Samen und Pflanzen AG | 2002 | Delley Samen und Pflanzen AG, 2013 (Switzerland) |
| 104 | Chilton | Great Britain | Deutsche Saatveredelung AG UK | 2013 | HGCA 2014-2015 (Great Britain) |
| 105 | Colonia | Germany | Limagrain GmbH | 2011 | Bundessortenamt, 2013 (Germany) |
| 106 | KWS Dacanto | Germany | KWS Lochow GmbH | 2011 | COBORU, 2014 (Poland) |
| 107 | Dekan | Germany | Lochow-Petkus | 2009 | Bundessortenamt, 2013 (Germany) |
| 108 | Desamo | Germany | Syngenta Seeds GmbH | 2009 | Bundessortenamt, 2013 (Germany) |
| 109 | Descartes | France | Secobra Recherches | 2011 | GEVES, 2013 (France) |
| 110 | Diamento | France | RAGT 2n | 2011 | GEVES, 2013 (France) |
| 111 | Diderot | France | Secobra Recherches | 2011 | GEVES, 2013 (France) |
| 112 | Discus | Germany | Pflanzenzucht SaKa GmbH&Co.KG | 2007 | Bundessortenamt, 2013 (Germany) |
| 113 | EDELRUN | Austria | Saatzucht LFS | 2013 | Österreichische Beschreibende Sortenliste, 2014 (Austria) |
| 114 | Edgar | Germany | Limagrain GmbH | 2010 | Bundessortenamt, 2013 (Germany) |
| 115 | Ehogold 770/09 | Austria | NA | NA | Landwirtschaftliche Fachschule Edelhof, 2014 (Austria) |
| 116 | Elixer | Germany | W. von Borries-Eckendorf GmbH & Co. KG | 2012 | COBORU, 2014 (Poland) |
| 117 | Eperon | France | Saaten Union Recherche | 2012 | GEVES, 2012 (France) |
| 118 | Eron | Austria | NA | NA | Saatzucht LFS, 2013 (Austria) |
| 119 | Etana | Czech Republic | Deutsche Saatveredelung AG | 1991 | Deutsche Saatveredelung AG, 2013 (Germany) |
| 120 | Evolution | Denmark | Sejet Planteforaedling | NA | HGCA 2014-2015 (Great Britain) |
| 121 | Famulus | Germany | Deutsche Saatveredelung AG | 2010 | Bundessortenamt, 2013 (Germany) |
| 122 | Fermi | Czech Republic | Florimond Desprez | 1961 | Florimond Desprez, 2013 (France) |
| 123 | Florett | Great Britain | NA | NA | Risser et al. (2011) |
| 124 | Florian | Germany | Nordsaat Saatzucht GmbH | 2010 | Bundessortenamt, 2013 (Germany) |
| 125 | Fructidor | France | Unisigma | 2013 | GEVES, 2013 (France) |
| 126 | Frument | Denmark | Sejet Planteforaedling | 2005 | Bundessortenamt, 2013 (Germany) |
| 127 | Gabrio | France | Syngenta Seeds GmbH | 2014 | GEVES, 2012 (France) |
| 128 | Glaucus | Germany | Strube Research GmbH & Co. KG | 2011 | Bundessortenamt, 2013 (Germany) |
| 129 | Gordian | Germany | Syngenta Seeds GmbH | 2009 | Bundessortenamt, 2013 (Germany) |
| 130 | Granamax | France | Agri Obtentions SA | 2013 | GEVES, 2013 (France) |
| 131 | Grapeli | France | Agri Obtentions SA | 2011 | GEVES, 2012 (France) |
| 132 | Heros | Denmark | Sejet Planteforaedling | 2012 | GEVES, 2012 (France) |
| 133 | Intro | France | RAGT 2n | 2011 | Bundessortenamt, 2013 (Germany) |
| 134 | Ionesco | France | Secobra Recherches | 2011 | GEVES, 2012 (France) |
| 135 | Jaceo | France | Syngenta | 2012 | GEVES, 2012 (France) |
| 136 | Joker | Germany | Deutsche Saatveredelung AG | 2012 | Bundessortenamt, 2013 (Germany) |
| 137 | Julius | Germany | KWS Lochow GmbH | 2008 | Bundessortenamt, 2013 (Germany) |
| 138 | Kalahari | Germany | Limagrain GmbH | 2010 | Bundessortenamt, 2013 (Germany) |
| 139 | Kantao | France | RAGT 2n | 2012 | GEVES, 2012 (France) |
| 140 | Kerubino | Austria | Saatzucht Schmid Landau | 2004 | Österreichische Beschreibende Sortenliste, 2014 (Austria) |
| 141 | KWS Erasmus | Germany | KWS Lochow GmbH | 2010 | Bundessortenamt, 2013 (Germany) |
| 142 | Lahertis | Germany | Strube Research GmbH & Co. KG | 2004 | Bundessortenamt, 2013 (Germany) |
| 143 | Lear | Germany | Limagrain GmbH | 2010 | Bundessortenamt, 2013 (Germany) |
| 144 | Lithium | France | KWS Momont SAS | 2013 | GEVES, 2013 (France) |
| 145 | MV Lucilla | Hungary | Prebázis Kft. | 2007 | Agrártudományi Kutatóközpont Mezőgazdasági Intézet, 2013 (Hungary) |
| 146 | Magnus | Germany | Engelen Büchling | 2000 | Bundessortenamt, 2014 (Germany) |
| 147 | Manager | Germany | Saatzucht Schweiger GbR | 2006 | Bundessortenamt, 2013 (Germany) |
| 148 | Mandub | Germany | NA | NA | Piaskowaska et al. (2021) |
| 149 | Mandragor | France | Unisigma | 2012 | GEVES, 2012 (France) |
| 150 | Marcopolo | France | RAGT 2n | 2012 | GEVES, 2012 (France) |
| 151 | Matheo | Germany | Deutsche Saatveredelung AG | 2010 | Bundessortenamt, 2014 (Germany) |
| 152 | Matrix | Germany | Deutsche Saatveredelung AG | 2010 | Bundessortenamt, 2013 (Germany) |
| 153 | Mazurka | Hungary | NA | 2003 | Kelm et al. (2012) |
| 154 | Memory | France | Secobra Recherches | 2013 | Bundessortenamt, 2014 (Germany) |
| 155 | Mentor | France | RAGT 2n | 2012 | Bundessortenamt, 2014 (Germany) |
| 156 | Nelson | Germany | Saatzucht Schweiger GbR | 2011 | Bundessortenamt, 2014 (Germany) |
| 157 | Nocibe | France | Syngenta Seeds GmbH | 2012 | GEVES, 2012 (France) |
| 158 | Oceano | France | RAGT 2n | 2012 | GEVES, 2012 (France) |
| 159 | Opal | Germany | Syngenta Seeds GmbH | 2011 | Bundessortenamt, 2014 (Germany) |
| 160 | Pamier | Germany | Lantmännen SW Seed GmbH | 2008 | Bundessortenamt, 2014 (Germany) |
| 161 | Pionier | France | Secobra Recherches | 2013 | Bundessortenamt, 2014 (Germany) |
| 162 | Primus | Germany | Deutsche Saatveredelung AG | 2009 | Bundessortenamt, 2014 (Germany) |
| 163 | Pueblo | France | RAGT 2n | 2012 | GEVES, 2012 (France) |
| 164 | Réciproc | France | Lemaire Deffontaines | 2013 | GEVES, 2013 (France) |
| 165 | Renan (RL 248) | France | Agri-Obtentions | 1993 | Florimond Desprez, 2013 (France) |
| 166 | RGT Ampiezzo | France | RAGT 2n | 2013 | GEVES, 2013 (France) |
| 167 | RGT Djoko | France | RAGT 2n | 2013 | GEVES, 2013 (France) |
| 168 | RGT Frenezio | France | RAGT 2n | 2013 | GEVES, 2013 (France) |
| 169 | RGT Kilimanjaro | France | RAGT 2n | 2013 | GEVES, 2013 (France) |
| 170 | Riband | Great Britain | NA | NA | Chartrain et al. (2004) |
| 171 | Rigi | Switzerland | Delley Seeds and Plants Ltd. | 2004 | Delley Samen und Pflanzen AG, 2013 (Switzerland) |
| 172 | Rumor | Germany | Dr. Hermann Strube | 2013 | Bundessortenamt, 2014 (Germany) |
| 173 | SALUTOS | Germany | Deutsche Saatveredelung AG | 2013 | Deutsche Saatveredelung AG, 2013 (Germany) |
| 174 | Samurai | Germany | Deutsche Saatveredelung AG | 2005 | Florimond Desprez, 2013 (France) |
| 175 | Scaro | Switzerland | Getreidezüchtung Peter Kunz | 2005 | Getreidezüchtung Peter Kunz, 2013 (Germany) |
| 176 | Schamane | Germany | Saatzucht Engelen Büchling e.K. | 2005 | Bundessortenamt, 2013 (Germany) |
| 177 | Sokrates | Germany | Saatzucht Engelen Büchling e.K. | 2001 | Bundessortenamt, 2013 (Germany) |
| 178 | Solognac | France | Caussade Semences | 2013 | GEVES, 2013 (France) |
| 179 | Sophytra | Netherlands | Limagrain Nederland B.V. (LG Europe-Research) | 2008 | Bundessortenamt, 2014 (Germany) |
| 180 | Starway | France | Semalliance | 2013 | GEVES, 2013 (France) |
| 181 | Syllon | France | Syngenta Seeds | 2013 | GEVES, 2013 (France) |
| 182 | Tabasco | Germany | W. von Borries-Eckendorf GmbH & Co. KG | 2008 | Bundessortenamt, 2014 (Germany) |
| 183 | Tengri | Switzerland | Getreidezüchtung Peter Kunz | 2007 | Getreidezüchtung Peter Kunz, 2013 (Germany) |
| 184 | Tentation | France | Lemaire Deffontaines | 2013 | GEVES, 2013 (France) |
| 185 | Terroir | France | Florimond Desprez | 2012 | GEVES, 2012 (France) |
| 186 | Thalys | France | Syngenta Seeds | 2012 | GEVES, 2012 (France) |
| 187 | Tobak | Germany | W. von Borries-Eckendorf GmbH & Co. KG | 2011 | Bundessortenamt, 2014 (Germany) |
| 188 | Tuareg | Germany | Nordsaat Saatzucht GmbH | 2005 | Risser et al.(2011) |
| 189 | Valdo | France | RAGT 2n | 2012 | GEVES, 2012 (France) |
| 190 | Waxy | Germany | Dieckmann GmbH & Co. KG | 2012 | Hodowla Roślin Strzelce Sp z o. o., Grupa IHAR |
| 191 | Winnetou | Germany | Saatzucht Firlbeck | 2002 | Bundessortenamt, 2014 (Germany) |
| 192 | Wiwa | Switzerland | Getreidezüchtung Peter Kunz | 2005 | Getreidezüchtung Peter Kunz, 2013 (Germany) |
| 193 | Xantippe | Denmark | Sejet Planteforaedling | 2011 | Bundessortenamt, 2014 (Germany) |
| 194 | Zappa | Germany | Ackermann Saatzucht GmbH & Co. KG | 2009 | Bundessortenamt, 2014 (Germany) |
| 195 | Zephyr | France | KWS Momont SAS | 2012 | GEVES, 2012 (France) |
| 196 | Zeppelin | Germany | Syngenta Seeds GmbH | 2012 | Bundessortenamt, 2014 (Germany) |
| 197 | Zobel | Germany | Syngenta Seeds GmbH | 2006 | Bundessortenamt, 2014 (Germany) |
| 198 | Bulgaria88 | Bulgaria | NA | NA | Adhikari et al. (2004a), Chartrain et al.(2005a) |
| 199 | Veranopolis | Brazil | NA | NA | Liu et al. (2013), Chartrain et al. (2005a) |
| 200 | Israel493 | Israel | NA | NA | Goodwin et al. (2015), Chartrain et al.(2005a) |
| 201 | Tadinia | USA | NA | NA | Adhikari et al. (2004b), Chartrain et al. (2005a) |
| 202 | Cs Synthetic (6x)7D | China/USA | NA | NA | Arraiano et al. (2001) |
| 203 | Flame | Great Britain | NA | NA | Brading et al. (2002), Chartrain et al. (2005a) |
| 204 | Estanzuela Federal | Uruguay | NA | NA | McCartney et al. (2003) |
| 205 | M6 synthetic (W-7984) | USA | NA | NA | Adhikari et al. (2003) |
| 206 | Courtot | France | Agri Obtentions SA | 2002 | Chartrain et al. (2009) |
| 207 | TE9111 | Portugal | NA | NA | Chartrain et al. (2005b) |
| 208 | Salamouni | Canada | NA | NA | KOMUGI (2021) |
| 209 | Arina | Switzerland | DSP | 2002 | Arraiano et al. (2007), Chartrain et al. (2005a) |
| 210 | M3 synthetic (W-7976) | USA | NA | NA | Tabib Ghaffary et al. (2012) |
| 211 | Liwilla | Poland | DANKO Hodowla Roślin sp. z o. o. | 1980 | Radecka-Janusik and Czembor (2014) |
| 212 | Solitar | Germany | NA | NA | Kelm et al. (2012) |
| 213 | Apache | France | Limagrain Europe | 1999 | Tabib Ghaffary et al. (2011) |
| 214 | Balance | France | NA | NA | Tabib Ghaffary et al. (2011) |
| 215 | Chinese Spring | China | NA | landrace | Brading et al. (2002), Chartrain et al. (2005a) |
| 216 | Taichung29 | Japan | NA | unknown | Tabib Ghaffary et al. (2011) |
| 217 | Begra | Poland | DANKO Hodowla Roślin sp. z o. o. | 1982 | Czembor et al. (2011), Radecka-Janusik and Czembor (2014) |

*NA – data not available

** Table reference list:

COBORU, Centralny Ośrodek Badania Odmian Roślin Uprawnych. 2014. Lista opisowa odmian roślin rolniczych – zbożowe. Słupia Wielka, pp. 76.

GEVES, Groupe d’Étude et de Contrôle des Variétés et des Semences. 2013. Céréales a paille. La Pouëze, No. 53, pp. 26.

Bundessortenamt. 2013. Beschreibende Sortenliste, Getreide, Mais, Ӧl- und Faserpflanzen, Leguminosen, Rüben, Zwischenfrüchte. Hannover, pp. 297.

Österreichische Beschreibende Sortenliste. 2014. https://www.baes.gv.at/zulassung/pflanzensorten/oesterreichische-sortenliste

HGCA 2014-2015, Recommendation Lists for cereals and oilseeds. https://ahdb.org.uk/rlarchive

John Innes Centre. 2013. https://www.jic.ac.uk/

ÚKZÚZ, Ústřední Kontrolní a Zkušební Ústav Zemědělský, Recommended Lists of plant varieties 2013. http://eagri.cz/public/web/en/ukzuz/portal/plant-varieties/publications/recommended-list-of-plant-varieties/archive-1/x2013/

Deutsche Saatveredelung AG. 2013. https://www.dsv-saaten.de/

Delley Samen und Pflanzen AG. 2013. https://www.dsp-delley.ch/de/

Landwirtschaftliche Fachschule Edelhof. 2014. https://www.lfs-edelhof.ac.at/

GEVES, Groupe d’Étude et de Contrôle des Variétés et des Semences. 2012. Céréales a paille. La Pouëze, No. 51, pp. 29.

Saatzucht LFS. 2013. https://saatzucht.edelhof.at/

Florimond Desprez. 2013. http://www.florimond-desprez.com

Risser P, Ebmeyer E, Korzun V, Hartl L, Miedaner T. Quantitative trait loci for adult-plant resistance to *Mycosphaerella graminicola* in two winter wheat populations. Phytopathology. 2011;101:1209–1216.

Agrártudományi Kutatóközpont Mezőgazdasági Intézet. 2013. http://mgi.atk.hu/hu

Bundessortenamt. 2014. Beschreibende Sortenliste, Getreide, Mais, Ӧl- und Faserpflanzen, Leguminosen, Rüben, Zwischenfrüchte. Hannover, pp. 297.

Piaskowska D, Piechota U, Radecka-Janusik M, Czembor P. QTL Mapping of Seedling and Adult Plant Resistance to Septoria Tritici Blotch in Winter Wheat cv. Mandub (*Triticum aestivum* L.). Agronomy. 2021; 11:1108.

Chartrain L, Brading PA, Widdowson JP, Brown JKM. Partial resistance to Septoria tritici blotch (*Mycosphaerella graminicola*) in the wheat cultivars Arina and Riband. Phytopathology. 2004;94:497–504.

Getreidezüchtung Peter Kunz. 2013. https://www.gzpk.ch/

Hodowla Roślin Strzelce Sp z o. o., Grupa IHAR. 2013. https://hr-strzelce.pl/

Adhikari TB, Yang X, Cavaletto JR, Hu X, Buechley G, Ohm HW, Shaner G, Goodwin SB. Molecular mapping of Stb1, a potentially durable gene for resistance to Septoria tritici blotch in wheat. Theor Appl Genet. 2004a;109:944–953.

Chartrain L, Berry ST, Brown JKM. Resistance of wheat line Kavkaz- K4500 L.6.A.4 to Septoria tritici blotch controlled by isolate-specific resistance genes. Phytopathology 2005a;95:664–671.

Liu Y, Zhang L, Thompson IA, Goodwin SB, Ohm HW. Molecular mapping re-locates the Stb2 gene for resistance to Septoria tritici blotch derived from cultivar Veranopolis on wheat chromosome 1BS. Euphytica. 2013;190:145–156.

Goodwin SB, Cavaletto JR, Hale IL, Thompson I, Xu SX, Adhikari T B, Dubcovsky J. A New Map Location of Gene Stb3 for Resistance to Septoria Tritici Blotch in Wheat. Crop Sci. 2015;55(1):35-43.

Adhikari TB, Cavaletto JR, Dubcovsky J, Gieco JO, Schlatter AR, Goodwin SB. Molecular mapping of the Stb4 gene for resistance to Septoria tritici blotch in wheat. Phytopathology. 2004b;94:1198–1206.

Arraiano LS, Worland AJ, Ellerbrook C, Brown JKM. Chromosomal location of a gene for resistance to Septoria tritici blotch (*Mycosphaerella graminicola*) in the hexaploid wheat ‘Synthetic 6x’. Theor Appl Genet. 2001;103:758–764.

Brading PA, Verstappen ECP, Kema GHJ, Brown JKM. A gene-for-gene relationship between wheat and *Mycosphaerella graminicola*, the Septoria tritici blotch pathogen. Phytopathology. 2002;92:439–445.

McCartney CA, Brûlé-Babel AL, Lamari L, Somers DJ. Chromosomal location of a race specific resistance gene to *Mycosphaerella graminicola* in the spring wheat ST6. Theor Appl Genet. 2003;107:1181–1186.

Adhikari TB, Anderson JM, Goodwin SB. Identification and molecular mapping of a gene in wheat conferring resistance to *Mycosphaerella graminicola*. Phytopathology 2003;93:1158-1164.

Chartrain L, Sourdille P, Bernard M, Brown JKM. Identification and location of Stb9, a gene for resistance to Septoria tritici blotch in wheat cultivars Courtot and Tonic. Plant Pathol. 2009;58: 547–555.

Chartrain L, Joaquim P, Berry ST, Arraiano LS, Azanza F, Brown JKM. Genetics of resistance to Septoria tritici blotch in the Portuguese wheat breeding line TE9111. Theor Appl Genet. 2005b;110:1138–1144.

KOMUGI, Wheat Genetic Resources Database, https://shigen.nig.ac.jp/wheat/komugi/genes/symbolClassList.jsp. Accessed December 2021.

Arraiano LS, Chartrain L, Bossolini E, Slatter HN, Keller B, Brown JKM. A gene in European wheat cultivars for resistance to an African isolate of *Mycosphaerella graminicola*. Plant Pathol. 2007;56:73–78.

Tabib Ghaffary SM, Faris JD, Friesen TL, Visser RGF, van der Lee TAJ, Robert O, Kema GHJ. New broad-spectrum resistance to Septoria tritici blotch derived from synthetic hexaploid wheat. Theor Appl Genet. 2012;124: 125–142.

Radecka-Janusik M, Czembor PC. Genetic mapping of quantitative trait loci (QTL) for resistance to septoria tritici blotch in a winter wheat cultivar Liwilla. Euphytica. 2014;200:109–125.

Kelm C, Tabib Ghaffary SM, Bruelheide H, Order MS, Miersch S, Weber WE, Kema GHJ, Saal B. The genetic architecture of seedling resistance to Septoria tritici blotch in the winter wheat doubled-haploid population Solitär x Mazurka. Mol Breed. 2012;29:813–830.

Tabib Ghaffary MT, Robert O, Laurent V, Lonnet P, Margale ́ E, van der Lee TA, Visser RG, Kema GH. Genetic analysis of resistance to Septoria tritici blotch in the French winter wheat cultivars Balance and Apache. Theor Appl Genet 2011;123:741–754.

Czembor PC, Radecka-Janusik M, Mańkowski D. Virulence spectrum of *Mycosphaerella graminicola* isolates on wheat genotypes carrying known resistance genes to Septoria tritici blotch. J Phytopathol. 2011;159:146–154.
